# Interactions between nascent proteins and the ribosome surface inhibit co-translational folding

**DOI:** 10.1038/s41557-021-00796-x

**Published:** 2021-10-14

**Authors:** Anaïs M. E. Cassaignau, Tomasz Włodarski, Sammy H. S. Chan, Lauren F. Woodburn, Ivana V. Bukvin, Julian O. Streit, Lisa D. Cabrita, Christopher A. Waudby, John Christodoulou

**Affiliations:** 1 Institute of Structural and Molecular Biology, University College London, London, UK; 2 Institute of Structural and Molecular Biology, Birkbeck College, University of London, London, UK

**Keywords:** Protein folding, Solution-state NMR, Computational models, Ribosome

## Abstract

Most proteins begin to fold during biosynthesis on the ribosome. It has been suggested that interactions between the emerging polypeptide and the ribosome surface might allow the ribosome itself to modulate co-translational folding. Here we combine protein engineering and NMR spectroscopy to characterize a series of interactions between the ribosome surface and unfolded nascent chains of the immunoglobulin-like FLN5 filamin domain. The strongest interactions are found for a C-terminal segment that is essential for folding, and we demonstrate quantitative agreement between the strength of this interaction and the energetics of the co-translational folding process itself. Mutations in this region that reduce the extent of binding result in a shift in the co-translational folding equilibrium towards the native state. Our results therefore demonstrate that a competition between folding and binding provides a simple, dynamic mechanism for the modulation of co-translational folding by the ribosome.

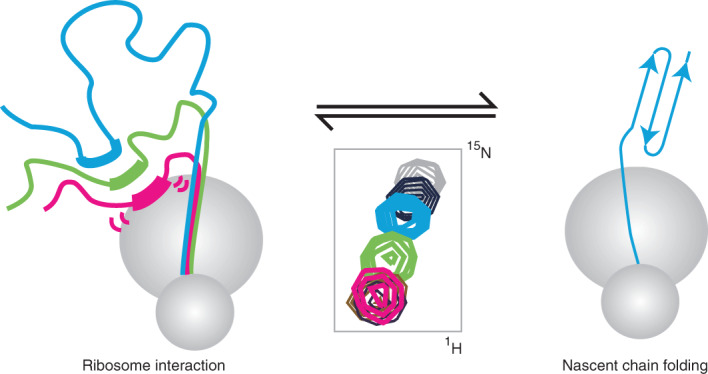

## Abstract (web summary)

During polypeptide biosynthesis, a strong interaction can occur between a segment of an emerged, disordered nascent protein and the ribosomal surface. Now, it has been shown that competition between this ribosomal binding and the folding energetics of an immunoglobulin-like domain modulates the mechanism of co-translational folding.

## Main

In the cell, most nascent polypeptide chains begin to fold during biosynthesis^1–3^. In many cases, co-translational folding increases the ability of a protein to efficiently attain its native structure^4–7^. This may in part be due to the ribosome modulating the conformational ensembles sampled by nascent chains^8–11^. The ribosome constrains disordered chains close to its charged surface^12^, and can promote the early formation of compact states during co-translational folding^13–16^. In general, nascent chains have been found to be destabilized when they are bound to the ribosome^5,17–19^. This could be linked to interactions between the disordered nascent chain and the ribosome surface^20^, some of which bear a partial electrostatic character^21,22^. Ribosome–nascent chain interactions have also been suggested to attenuate co-translational folding rates^23^, and to compete with co-translational assembly between a nascent chain and its binding partner^24^. Collectively, these studies point clearly towards a role for the ribosome in shaping the onset of co-translational folding^3^. However, due in large part to the technical difficulty of measuring the intramolecular equilibria associated with ribosome–nascent chain interactions, a link between the energetics of ribosome interactions and co-translational folding outcomes has not yet been established in quantitative terms.

**Fig. 1:**
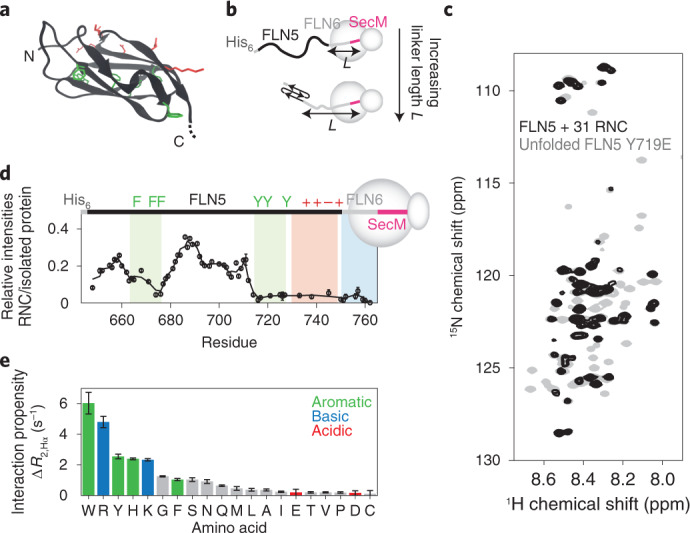
Identifying interactions of FLN5 RNCs with the ribosome surface. **a**, Cartoon representation of the FLN5 structure (PDB: 1QFH^48^). Aromatic (green) and surface-exposed (red) residues that have been mutated in this study are shown using stick representations. **b**, Design of FLN5+*L* RNC constructs, where *L* indicates the linker length, comprising a fragment of the following FLN6 domain and the 17-amino-acid SecM arrest peptide^20^. **c**, ^1^H,^15^N SOFAST-HMQC spectrum of a FLN5+31 RNC (blue) overlaid with that of the isolated, unfolded FLN5 Y719E variant (grey) (283 K, 950 MHz). Contour levels of the RNC spectrum are 3.8-fold lower than for isolated Y719E. **d**, Intensities of FLN5+31 RNC resonances relative to the isolated, unfolded FLN5 Y719E variant. A three-point moving average is shown. Shading indicates the approximate location of ribosome interaction sites characterized in this study. **e**, Transferred transverse ^1^H NMR relaxation measurements of the interaction of free amino acids with purified 70S ribosomes (283 K, 700 MHz). All error bars indicate standard errors derived from the spectral noise. Source data

In this article we study the co-translational folding of FLN5, a 105 residue immunoglobulin-like domain from the tandem repeat protein filamin (Fig. 1a), using SecM-arrested ribosome–nascent chain complexes (RNCs) in which FLN5 is tethered to the ribosome via variable lengths of the following domain, FLN6 (Fig. 1b)^20,25^. Measurements of the accessibility of a C-terminal cysteine to covalent modification by PEG-maleimide (PEGylation) showed that the entire FLN5 domain emerges beyond the ribosome exit tunnel for linkers comprising at least 31 residues^20^ (FLN5+31). However, NMR observations demonstrated that FLN5 remains partially unfolded until the linker extends beyond 42 residues. This offset between the emergence of FLN5 and its folding suggests that the ribosome has a destabilizing effect on co-translational folding, which we speculated could be due to interactions between the unfolded nascent chain and the ribosome surface^20^. We use NMR spectroscopy together with protein engineering, molecular dynamics simulations and PEGylation measurements of nascent chain stability to identify a series of interaction sites on the nascent chain of varying affinities, and their impact on co-translational folding.

## Results

### Identification of regions of FLN5 RNCs interacting with the ribosome surface

**Extended Data Fig. 1:**
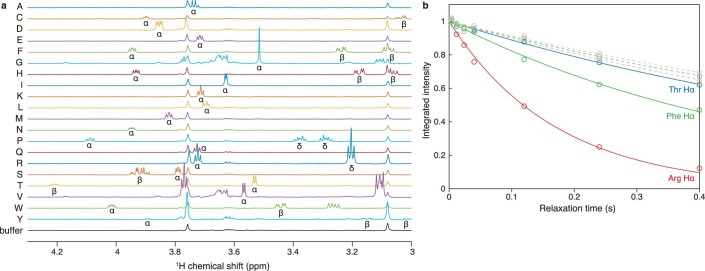
Interaction of free amino acid with empty 70S ribosomes. (**a**) Hα region of 1D ^1^H NMR spectra of isolated amino acids (700 MHz, 283 K), showing atom assignments. (**b**) Representative ^1^H *R*_2_ relaxation measurements for the indicated amino acid resonances in the presence (solid lines) and absence (dashed lines) of 1 µM 70S ribosomes. Source data

Since the FLN5+31 RNC represents the first point during protein biosynthesis when the entire sequence is available for folding, we have used this biosynthetic snapshot as a starting point to examine how the ribosome might modulate the dynamic properties of a nascent chain. Previously, a comparison of the 2D ^1^H,^15^N NMR correlation spectrum of a FLN5+31 RNC against an isolated unfolded variant, Y719E, revealed site-selective line broadening that we interpreted as evidence of such interactions (Fig. 1c,d). From this, we have identified three main regions for investigation here. First, two clusters of aromatic residues, located within the core of the folded domain (Fig. 1a), were identified as displaying substantial broadening; these are referred to here as ‘F_3_’ (residues F665–F675) and ‘Y_3_’ (residues Y715–Y727) sites (Fig. 1d, green). Second, strong line broadenings were observed in the mildly basic C-terminal region of FLN5 (residues N728–C747, the ‘C-terminal segment’ (Fig. 1d, red). These observations correlate well with measurements of the interaction between individual amino acids and purified 70S ribosomes, which are strongest for aromatic and basic side chains (Fig. 1e and Extended Data Fig. 1). Third, resonances of the FLN6 tether are broadened to varying extents across FLN5 RNCs of increasing lengths, which, in part, relates to its occlusion within the ribosomal exit tunnel^20^ (Fig. 1d, cyan).

#### Interactions of F_3_ and Y_3_ aromatic clusters with the ribosome surface

**Fig. 2:**
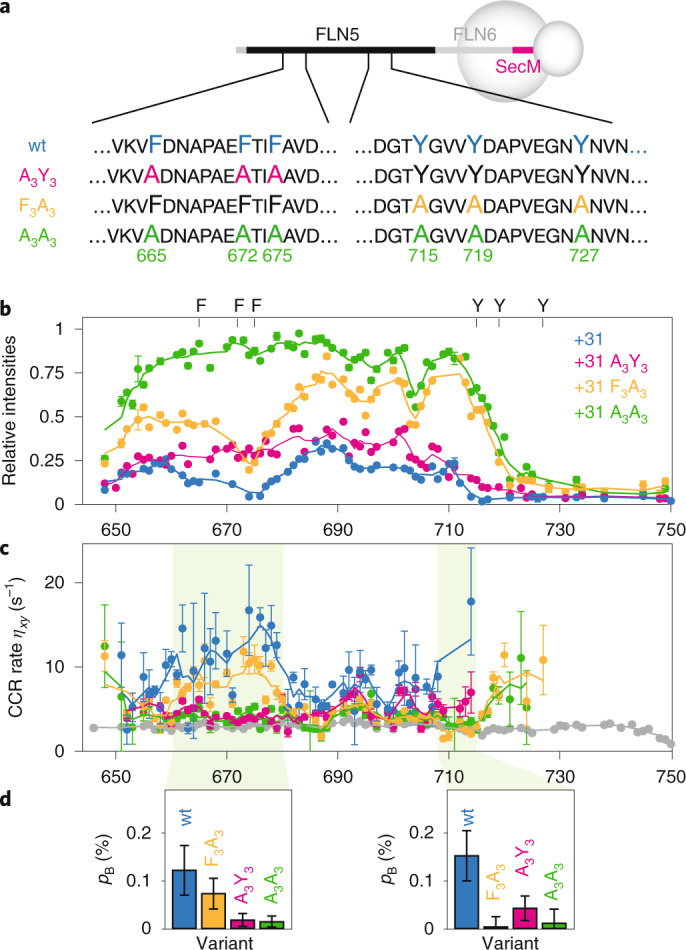
Quantification of interactions of aromatic clusters with the ribosome surface. **a**, Design of aromatic to alanine FLN5 variants. **b**, ^1^H,^15^N SOFAST-HMQC resonance intensities (950 MHz, 283 K) of FLN5+31 RNC variants relative to the corresponding isolated unfolded state. **c**, Transverse cross-correlated relaxation rates (950 MHz, 283 K) of FLN5+31 RNC variants (colours as in **b**), and isolated FLN5 A_3_A_3_ (grey). Errors were derived from the spectral noise for F_3_A_3_, A_3_Y_3_ and A_3_A_3_ variants, while the mean and standard error from three biological repeats are shown for the wt. Three-point moving averages are shown. **d**, Bound state populations, *p*_B_, of the highlighted nascent chain segments (residues 660–680 and residues 708–716) determined by analysis of transferred cross-correlated relaxation. Error bars represent the standard deviation of residues within these segments. Source data

**Extended Data Fig. 2:**
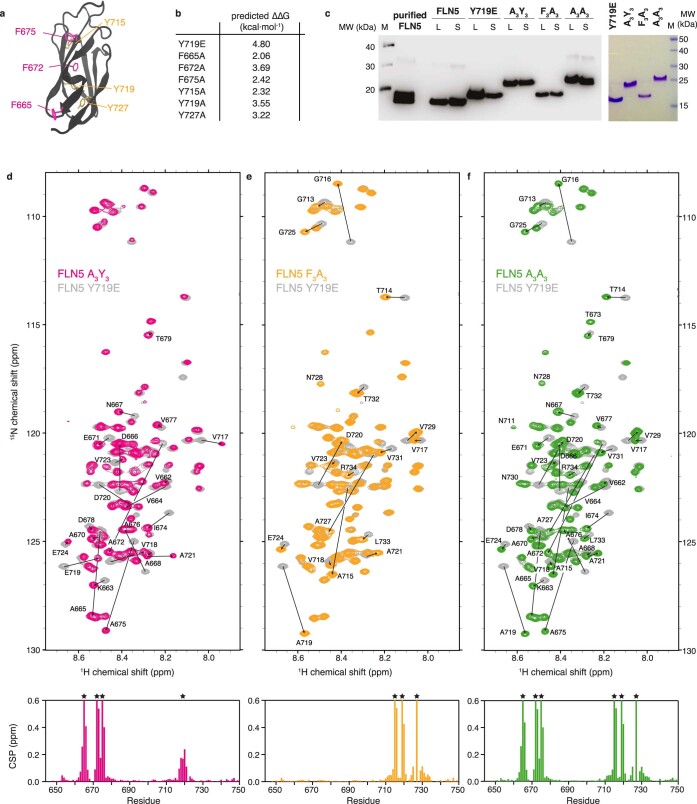
Biochemical and NMR analysis of isolated aromatic variants. (**a**) Aromatic residues that have been mutated in this study mapped onto the PDB structure of FLN5 (PDB 1QFH). (**b**) POPMUSIC stability predictions of the FLN5 aromatic mutations^65^. (**c**) Western blot (anti-His tag) of an expression text of FLN5, FLN5 Y719E, FLN5 A_3_Y_3_, FLN5 F_3_A_3_ and FLN5 A_3_A_3_ proteins (L: total lysate, S: soluble lysate) and Coomassie stained SDS PAGE gel of purified FLN5 Y719E, FLN5 A_3_Y_3_, FLN5 F_3_A_3_ and FLN5 A_3_A_3_ proteins. (**d**–**f**) ^1^H,^15^N correlation spectrum (283 K) of FLN5 Y719E overlaid with that of (d) FLN5 A_3_Y_3_, (e) FLN5 F_3_A_3_ and (f) FLN5 A_3_A_3_, with assignments of the shifted resonances indicated. Combined amide chemical shift perturbations, ${\Delta}\delta = \sqrt {{\Delta}\delta _H^2 + \left( {{\Delta}\delta _N/5} \right)^2}$, are plotted below. The mutation sites relative to FLN5 Y719E have been indicated with star symbols on the CSP plot. Source data

**Extended Data Fig. 3:**
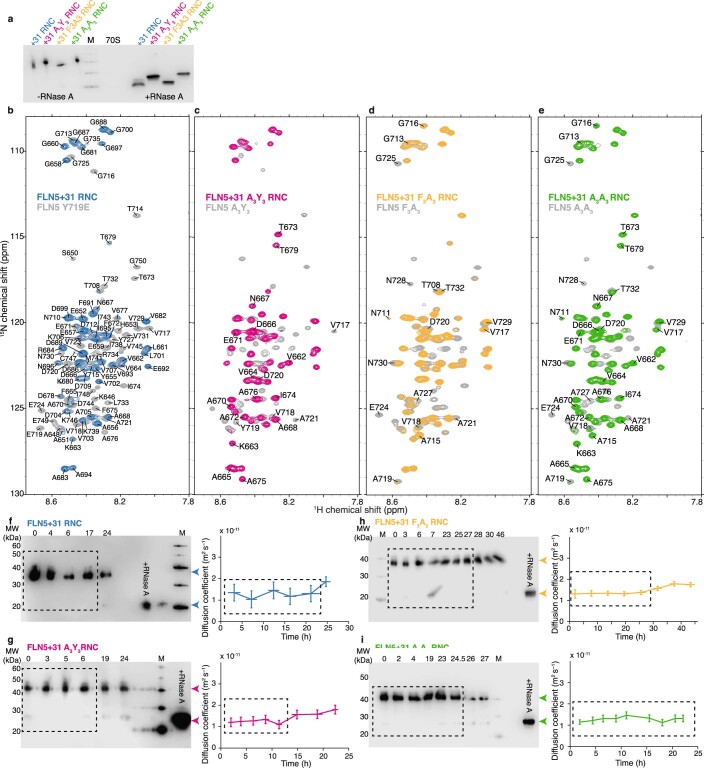
Biochemical and NMR analysis of aromatic RNC variants. (**a**) Anti-His tag western blot of FLN5+31 wt, A_3_Y_3_, F_3_A_3_ and A_3_A_3_ RNCs (10 pmol each) purified for NMR studies, in their tRNA-bound and released forms. The migration patterns on SDS page reflect those of the isolated mutant proteins (cf. Extended Data Fig. 2). (**b**–**e**) ^1^H,^15^N SOFAST-HMQC spectra (283 K, 950 MHz) of (b) FLN5+31, (c) FLN5+31 A_3_Y_3_, (d) FLN5+31 F_3_A_3_ and (e) FLN5+31 A_3_A_3_ RNCs against the corresponding isolated, unfolded FLN5 variants, with assignments. (**f**–**i**) Assessment of the integrity of ^15^N-labelled RNCs^25^: Anti-His western blots and translational diffusion coefficients measured using ^15^N SORDID experiments are shown. The timeframes during which nascent chains have been assessed as being intact and used for analysis are indicated (dashed boxes). Vertical error bars represent standard errors derived from the spectral noise; horizontal bars (where applicable) indicate the acquisition periods of diffusion measurements. Source data

We prepared a series of constructs, termed F_3_A_3_, A_3_Y_3_ and A_3_A_3_, in which the clusters of aromatic residues were mutated to alanine (Fig. 2a and Extended Data Fig. 2). The close overlay between ^1^H,^15^N NMR spectra of the isolated variants and disordered FLN5 indicates they are natively unfolded, as expected given large changes to the hydrophobic core (Extended Data Fig. 2c–f). On the ribosome, the ^1^H,^15^N correlation spectra of the corresponding FLN5+31 RNC variants showed substantial increases in cross-peak intensities relative to wild-type (wt) FLN5+31 RNC, broadly localized to the alanine mutation sites (Fig. 2b and Extended Data Fig. 3). Residues between A665 and G700 within the FLN5+31 A_3_A_3_ RNC variant reached a relative intensity close to 1, indicating that the mobility of this segment of the nascent chain is comparable to that of the isolated unfolded protein. These data demonstrate that the aromatic residues mediate a large proportion of the interactions at these sites. By contrast, in all variants the C-terminal segment of FLN5 (N728–C747) remained broadened beyond detection. This suggests additional interactions within this nascent chain segment (Fig. 2b).

**Extended Data Fig. 4:**
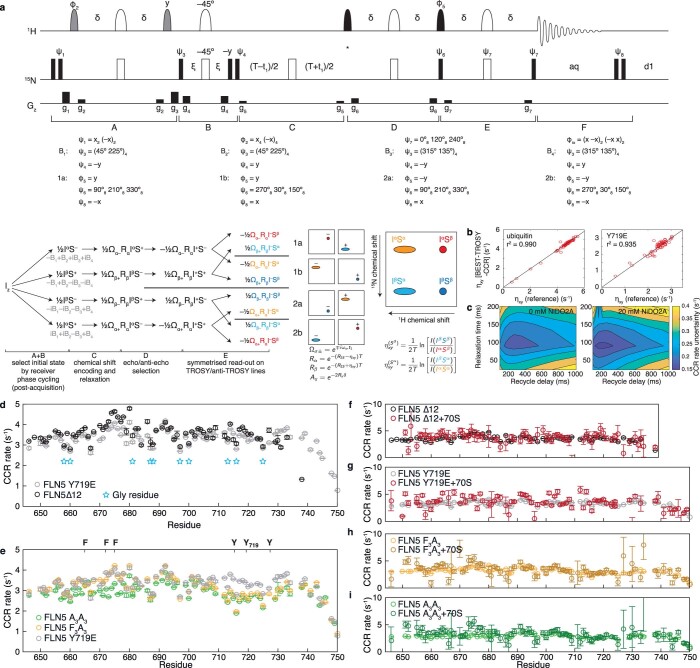
The BEST-TROSY-CCR experiment and application to isolated FLN5 variants. (**a**) Pulse sequence and processing scheme for the BEST-TROSY-CCR experiment. Solid coloring indicates 90° pulses; empty shapes indicate 180° pulses. ^1^H pulse lengths are calculated for application at an offset of 8.2 ppm (950 MHz). Gray ^1^H 90° pulses are Pc9_4_90 pulses (1958 µs), solid ^1^H 90° shapes indicate Eburp2 pulses (1251 µs, * indicates time reversed pulse), and ^1^H 180° shapes indicate Reburp pulses (1432 µs). ^15^N pulses are applied as high-power rectangular pulses. 16 subspectra are acquired, corresponding to the possible combinations of the phase programs B_1_–B_4_ and the phase programs 1a,1b,2a and 2b as indicated. All pulses are applied with a phase of x (0°) unless otherwise indicated. ^15^N phases are shown as spin dynamical phases and must be modified for application on Bruker spectrometers^66^. Gradients are applied as SMSQ shapes: *g*_1_ (31.4%, 1 ms), *g*_2_ (23%, 300 µs), *g*_3_ (21%, 1 ms), *g*_4_ (31%, 300 µs), *g*_5_ (11%, 1 ms), *g*_6_ (16.7%, 300 µs), *g*_7_ (45%, 300 µs). (**b**) Comparison of CCR rates measured for ubiquitin and FLN5 Y719E using our pulse sequence (111 ms relaxation delay, 283 K) against a reference measurement using symmetric recombination^67^. (**c**) Sensitivity enhancement on the CCR measurements of FLN5 Y719E (283 K, 950 MHz) obtained using the PLRE agent NiDO2A, an inert Ni^II^ chelate. (**d**) CCR rates of FLN5 Y719E and FLN5∆12. Blue stars indicate Gly residues, which on average have lower CCR rates. (**e**) CCR rates of FLN5 Y719E, FLN5 F_3_A_3_ and FLN5 A_3_A_3_. (**f**–**i**) Comparison of CCR rates of FLN5 variants at 10 µM, in presence and absence of an equimolar concentration of 70 S ribosomes: (f) FLN5∆12, (g) FLN5 Y719E, (h) FLN5 F_3_A_3_ and (i) FLN5 A_3_A_3_. All errors were derived from the spectral noise. Source data

To measure the strength of these aromatic interactions, we developed and acquired sensitivity-optimized measurements of the transverse cross-correlated relaxation (CCR) rate (Extended Data Fig. 4). Interactions of nascent chain segments with the ribosome surface will result in transferred relaxation, and thus an increase in the CCR rate, Δ*η*_*xy*_, relative to the isolated unfolded state. The increase, ${\Delta}\eta _{xy} = p_{\mathrm{B}}\eta _{xy}^{\mathrm{bound}}$, is proportional to the bound fraction *p*_B_, and to the CCR rate of the bound state, $\eta _{xy}^{\mathrm{bound}}$. Based on the known rotational correlation time (*τ*_C_) of the ribosome^26^, $\eta _{xy}^{\mathrm{bound}}$ is estimated to be ∼7,000 s^−1^.

CCR measurements were acquired for FLN5+31 wt, F_3_A_3_, A_3_Y_3_ and A_3_A_3_ RNCs, and the corresponding isolated proteins (Fig. 2c and Extended Data Fig. 4). In general, we observed that increased CCR rates were associated with reduced resonance intensities. In the wt RNCs, increases in *η*_*xy*_ of ∼15 s^−1^ were measured around the aromatic F_3_ cluster. Within the Y_3_ site, residues beyond T714 were broadened beyond detection in wt and A_3_Y_3_ RNCs, but examination of flanking residues indicates that *η*_*xy*_ probably increases substantially beyond 15 s^−1^. Increased relaxation was also observed for residues at the N terminus, which we ascribe to an interaction of the 6xHis tag as previously observed in α-synuclein RNCs^21^.

We observe some evidence of cooperativity between the F_3_ and Y_3_ clusters, both by comparison of resonance intensities and of CCR rates: the elimination of one cluster leads to a small reduction in the interaction of the neighbouring cluster (Fig. 2b,d). However, in quantitative terms the extent of interaction is weak in all cases. Within the F_3_ cluster, observed increases in CCR rates correspond to ribosome-bound nascent chain populations of ∼0.1% (Fig. 2d). While resonances for the Y_3_ cluster were strongly broadened in wt and A_3_Y_3_, only allowing for a partial quantitation of CCR rates, the ribosome-bound population between residues 708 and 716 also neared ∼0.1%. Although these estimates assume a rigid bound state, even substantial flexibility that results in an order of magnitude decrease in $\eta _{xy}^{\mathrm{bound}}$ would indicate a bound population of only a few percent. Such weak interactions within these regions are not sufficient to perturb the co-translational folding process.

#### Interaction of the C-terminal segment with the ribosome surface

**Fig. 3:**
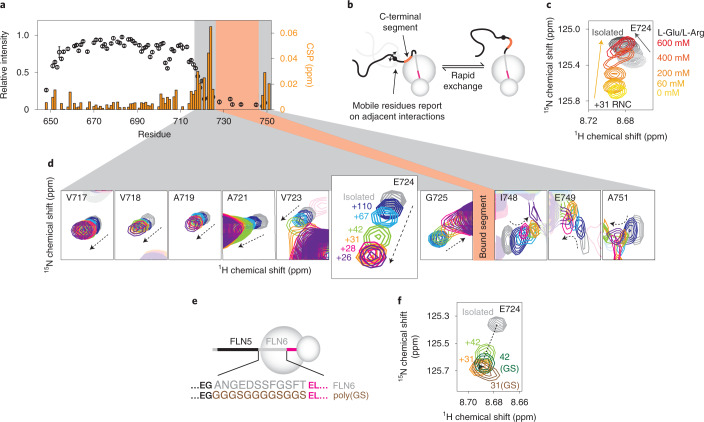
A ribosome-binding site in a C-terminal segment of FLN5. **a**, Resonance intensities (circles) and CSPs (bars) for the FLN5+31 A_3_A_3_ RNC relative to isolated A_3_A_3_. **b**, A model for the C-terminal segment in an equilibrium between ribosome-bound and unbound states. **c**, Magnified view of the E724 resonance in ^1^H,^15^N SOFAST-HMQC spectra of an FLN5+31 A_3_A_3_ RNC (yellow-red) and isolated FLN5 A_3_A_3_ (grey), shown at increasing ionic strengths (0–600 mM Glu/Arg). **d**, ^1^H,^15^N SOFAST-HMQC spectra showing isolated FLN5 A_3_A_3_ and FLN5 A_3_A_3_ RNC resonances, with linker lengths as indicated for the E724 resonance. Intensities have been rescaled for clarity. The location of the bound nascent chain segment is indicated with red shading. **e**, Design of poly(GS) linker RNCs. **f**, ^1^H,^15^N SOFAST-HMQC spectra of FLN5 A_3_A_3_ RNCs, centred on the E724 resonance and containing the native FLN6 or poly(GS) linker sequences as indicated. All data were acquired at 950 MHz, 283 K. Source data

**Extended Data Fig. 5:**
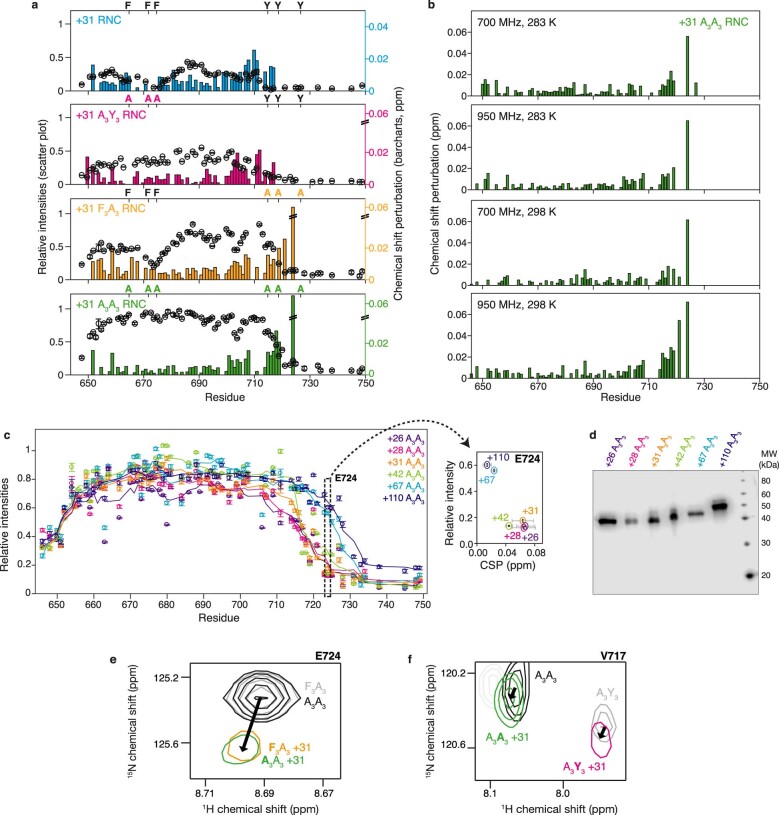
CSPs of FLN5 RNCs against isolated FLN5 variants, and comparison of chemical shift perturbations in the C-terminal region of FLN5 RNCs between aromatic cluster variants. (**a**) Relative intensities (LH axis) of FLN5+31, FLN5+31 A_3_Y_3_, FLN5+31 F_3_A_3_ and FLN5+31 A_3_A_3_ RNCs (283 K) and chemical shift perturbations (RH axis, ${\Delta}\delta = \sqrt {{\Delta}\delta _H^2 + \left( {{\Delta}\delta _N/5} \right)^2}$)), relative to their corresponding isolated, unfolded FLN5 variants. (**b**) Chemical shift perturbations of FLN5+31 A_3_A_3_ RNC relative to isolated FLN5 A_3_A_3_, at fields and temperatures as indicated. (**c**) Relative intensities of FLN5+26, 28, 31, 42, 67, 110 A_3_A_3_ RNCs relative to isolated FLN5 A_3_A_3_ (283 K, 950 MHz). inset: Intensities and combined amide chemical shift perturbations (${\Delta}\delta = \left[ {{\Delta}\delta _H^2 + \left( {{\Delta}\delta _N/5} \right)^2} \right]^{1/2}$) of the E724 amide resonance observed relative to isolated FLN5 A_3_A_3_ in ^1^H,^15^N SOFAST-HMQC spectra of FLN5 A_3_A_3_ RNCs, with varying linker lengths as indicated. (**d**) Anti-His tag western blot of FLN5+26, 28, 31, 42, 67, 110 A_3_A_3_ RNCs purified for NMR studies, in their tRNA-bound form. (**e**) Comparison of C-terminal chemical shift perturbations between A_3_A_3_ and F_3_A_3_ variants. The difference in perturbations between A_3_A_3_ and F_3_A_3_ variants was 0.009 ± 0.050 ppm (^15^N) and 0.002 ± 0.006 ppm (^1^H). (**f**) Comparison of C-terminal chemical shift perturbations between A_3_A_3_ and A_3_Y_3_ variants. Due to chemical shift perturbations between the A_3_A_3_ and A_3_Y_3_ variants, and the reduced intensity of C-terminal residues in the A_3_Y_3_ variant **(**Fig. 2b**)**, E724 cannot be resolved in the A_3_Y_3_ spectrum and residue V717 is analyzed instead. The difference in perturbations between A_3_A_3_ and A_3_Y_3_ variants was 0.060 ± 0.044 ppm (^15^N) and 0.001 ± 0.006 ppm (^1^H). Based on these negligible differences, mutations in the F_3_ and Y_3_ clusters do not seem to affect the CSP which report on the ribosome interaction of the C-terminal segment (residues N728 to C747). Source data

We next sought to investigate the interaction of the C-terminal segment (N728–C747) (Fig. 1d). The elimination of the Y_3_ cluster in the F_3_A_3_ and A_3_A_3_ variants resulted in newly observable resonances flanking this segment. Careful inspection revealed that small chemical shift perturbations (CSPs) were observed for these resonances (V717–G725, encompassing part of the Y_3_ region), relative to the isolated protein, and that their magnitude increased towards the (unobserved) ribosome-binding segment (Fig. 3a,b and Extended Data Fig. 5). Similarly, CSPs were observed at the C-terminal end of the binding segment between I748 and A751. Focusing on the A_3_A_3_ variant, we found that these CSPs were substantially reduced at high ionic strength (Fig. 3c), suggesting that they are associated with an interaction mediated at least in part by an electrostatic contribution.

We then explored the effect of the RNC linker length on the observed CSPs. We hypothesized that a shorter RNC would experience a higher effective ribosome concentration^20^ and therefore modulate the extent of binding. Indeed, as the linker length is increased from 26 to 110 amino acids, CSPs were observed to decrease (Fig. 3d) while resonance intensities increased (Extended Data Fig. 5). The CSPs at different lengths were collinear, indicating that changes in the ^1^H and ^15^N chemical shifts were strongly correlated (Fig. 3d). These collinear, correlated CSPs are an unambiguous indication that the C-terminal segment is rapidly exchanging between a ribosome-bound and free state, such that the observed chemical shift reflects a population-weighted average of unbound and bound states^27^. Resonances of the C-terminal residues I749–A751 showed deviations from collinearity at short linker lengths, which we attribute to proximity to the exit tunnel. For this reason, they have been excluded from further analysis. Together, these observations provide compelling evidence for a strong ribosome interaction involving nascent chain residues between Y727 and C747.

Alanine mutations within the F_3_ and Y_3_ clusters did not substantially perturb the observed C-terminal CSPs, indicating that there is no detectable cooperativity between interactions of these aromatic clusters and the C-terminal segment (Extended Data Fig. 5). Furthermore, no modulations in chemical shifts or intensities were observed upon varying magnetic field strength (Extended Data Fig. 5). Given the fast chemical exchange behaviour observed, we can infer from the largest frequency difference, Δ*ν*, of 30 Hz (for the ^15^N resonance of E724 at 22.3 T) that the dissociation of the bound state is rapid, with a lifetime much less than 3 ms (*τ* ≪ 1/4πΔ*ν*, that is, *k*_off_ ≈ *τ*^−1^ ≫ 360 s^−1^).

#### Impact of the linker sequence composition on FLN5 interactions

**Extended Data Fig. 6:**
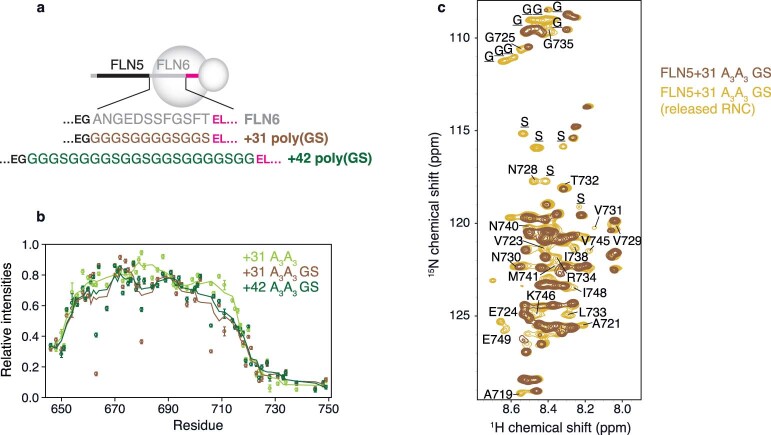
Analysis of FLN5-poly(GS) RNCs. (**a**) Design of GS linker RNCs. (**b**) ^1^H,^15^N SOFAST-HMQC resonances intensities for FLN5+31 A_3_A_3_, +31 A_3_A_3_ GS and +42 A_3_A_3_ GS RNCs relative to isolated FLN5 A_3_A_3_. (**c**) Overlay of a ^1^H,^15^N spectrum of FLN5+31 A_3_A_3_ GS RNC and a ^1^H,^15^N spectrum of the same RNC after treatment with 20 mM EDTA to induce nascent chain release. The residues only observable in EDTA-treated FLN5+31 A_3_A_3_ GS RNC are assigned, including resonances marked ‘G’ and ‘S’ which we attribute to the poly(GS) linker. Source data

We investigated the influence of the FLN6 linker on FLN5–ribosome interactions. We previously reported that replacing all FLN6 residues with a poly(GS) sequence (Fig. 3e) did not perturb co-translational folding equilibria^20^. Here we find that substituting poly(GS) linkers within A_3_A_3_ RNCs led to only small shifts in the CSPs and resonance intensities in the C-terminal segment, indicating that the extent of binding is not greatly perturbed (Fig. 3f and Extended Data Fig. 6), and suggesting little impact of the nature of the tether on the co-translational folding of FLN5.

### Quantification and molecular modelling of the C-terminal segment interaction

**Fig. 4:**
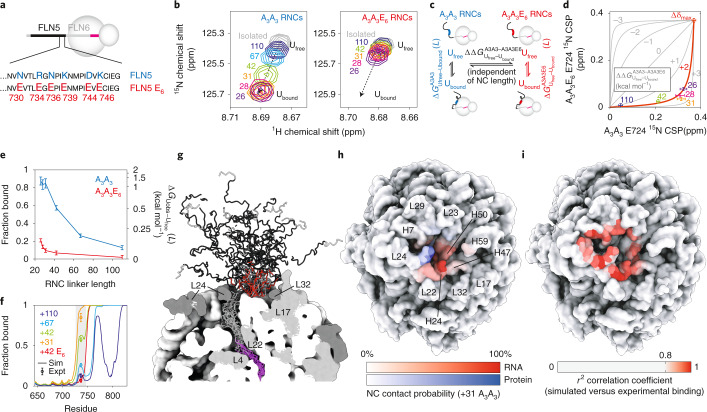
Quantification and molecular modelling of interactions between the FLN5 C-terminal region and the ribosome surface. **a**, Design of the FLN5 E_6_ variant. **b**, ^1^H,^15^N SOFAST-HMQC spectra (283 K, 950 MHz) showing the E724 resonance within FLN5 A_3_A_3_ RNCs and FLN5 A_3_A_3_E_6_ RNCs (linker lengths as indicated). Intensities have been rescaled for clarity. **c**, Equilibria underlying the joint analysis of A_3_A_3_ and A_3_A_3_E_6_ nascent chain–ribosome interactions. **d**, Correlation plot of E724 ^15^N chemical shift perturbations in A_3_A_3_ versus A_3_A_3_E_6_ RNCs, with linker lengths as indicated. A global fit across multiple resonances (Extended Data Fig. 7) is shown (red line) from which ${\Delta}{\Delta}G_{{\mathrm{U}}_{{\mathrm{free}}} - {\mathrm{U}}_{{\mathrm{bound}}}}^{{\mathrm{A3A3}} - {\mathrm{A3A3E6}}}$ is derived. **e**, Binding of the FLN5 C-terminal region as a function of linker length. The right-hand axis shows the predicted perturbation to the observed co-translational folding equilibrium in folding-capable RNCs (equation (2)). **f**, Interaction propensities of unfolded FLN5 nascent chain residues with the ribosome surface based on coarse-grained molecular dynamics simulations. Markers with error bars indicate bound fractions of the C-terminal region (shaded) determined experimentally (Fig. 4e). **g**, A structural ensemble of the FLN5+67 nascent chain determined through molecular dynamics simulations. The nascent chain is coloured as in **a**, with the interacting segment highlighted in red. Ribosomal RNA and proteins are coloured white and grey, respectively. **h**, Contact probabilities between ribosome protein or RNA residues and the C-terminal region of the FLN5+31 A_3_A_3_ nascent chain, determined through coarse-grained molecular dynamics simulations. **i**, Correlation across multiple nascent chain lengths between nascent chain–ribosome contact probabilities and the experimentally determined binding between the ribosome and the C-terminal region of FLN5 nascent chains. Source data

**Extended Data Fig. 7:**
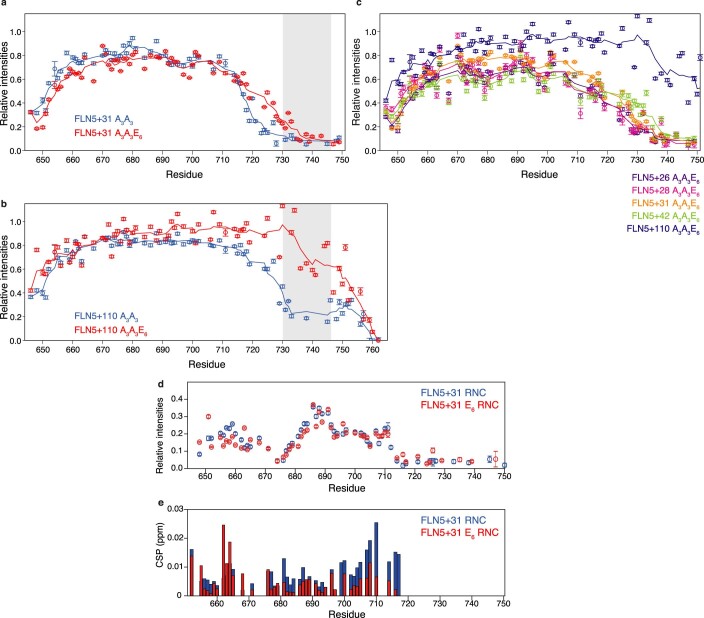
Characterization of FLN5 E6 variants. (**a**) ^1^H,^15^N SOFAST-HMQC resonance intensities of FLN5+31 A_3_A_3_ RNC relative to isolated FLN5 A_3_A_3_, and of FLN5+31 A_3_A_3_E_6_ RNC relative to isolated FLN5 A_3_A_3_E_6_ (283 K, 950 MHz). The region between residues 730–746 is highlighted in light grey. (**b**) Same as in (a), except for FLN5+110 A_3_A_3_ and A_3_A_3_E_6_ RNCs. (**c**) ^1^H,^15^N SOFAST-HMQC resonance intensities of FLN5+26, 28, 31, 42, 110 A_3_A_3_ E_6_ RNCs relative to isolated FLN5 A_3_A_3_E_6_ (283 K, 950 MHz). All errors were derived from the spectral noise. (**d**) Relative intensities of FLN5+31 and FLN5+31 E_6_ RNCs (283 K) relative to their corresponding isolated, unfolded FLN5 variants. (**e**) Chemical shift perturbations (${\Delta}\delta = \sqrt {{\Delta}\delta _H^2 + \left( {{\Delta}\delta _N/5} \right)^2}$)) of FLN5+31 and FLN5+31 E_6_ RNCs (283 K) relative to their corresponding isolated, unfolded FLN5 variants. Source data

Having identified a ribosome interaction site in the C-terminal segment, we next examined its molecular basis. Although the C-terminal segment does not contain aromatic residues, it contains three basic side chains (R734, K739, K746) that we hypothesized would contribute towards the electrostatic interactions (Fig. 1e), given the reduced CSPs observed at increased ionic strength (Fig. 3c). To explore this, we designed a FLN5 construct, A_3_A_3_E_6_, in which six residues (surface-exposed within the folded state) were replaced with acidic glutamate residues, thus reversing the net charge within this segment from +1 to −6 (Figs. 1a and 4a). The E_6_ mutations were found to greatly reduce the magnitude of CSPs in the C-terminal segment (Fig. 4b), indicating that these mutations reduce the affinity of the segment for the ribosome surface. However, some CSPs and line broadening persist (Extended Data Fig. 7), suggesting that the binding interaction of this region is not abrogated completely. Similar reductions in CSPs were also observed between FLN5+31 wt and E6 RNCs, although due to the increased line broadening fewer resonances adjacent to the interacting segment could be resolved (Extended Data Fig. 7).

**Extended Data Fig. 8:**
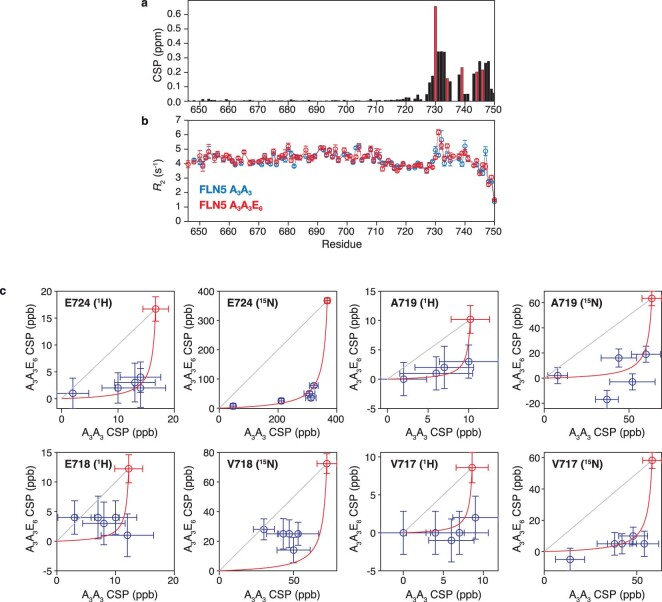
Global analysis of FLN5 A3A3 and A3A3E6 chemical shift perturbations. (**a**) Chemical shift perturbations of FLN5 A_3_A_3_ *vs*. FLN5 A_3_A_3_E_6_ (${\Delta}\delta = \sqrt {{\Delta}\delta _H^2 + \left( {{\Delta}\delta _N/5} \right)^2}$)). E_6_ mutation sides are indicated using red bar charts. (**b**) *T*_2_ relaxation measurements of isolated FLN5 A_3_A_3_ and FLN5 A_3_A_3_E_6._ (**c**) Correlation plots of ^1^H and ^15^N chemical shift perturbations in +26, +28, +31, +42 and +110 A_3_A_3_/A_3_A_3_E_6_ RNCs for residues as indicated (283 K, 950 MHz). A global fit of ${\Delta}{\Delta}G_{Ubound - Ufree}^{A3A3 - A3A3E6}$ is shown (red line) and red markers indicate the fitted values of Δ*δ*_*max*_ (χ^2^ = 70.17, dof = 66, χ^2^/ν = 1.06). Source data

To quantify the effect of the E_6_ mutations on the binding interaction, we have analysed further the observed CSPs, which reflect a population-weighted average of unbound and bound states. These residues provide a convenient ‘ruler’ to compare the interactions of different nascent chains with the ribosome. However, to determine the absolute amount of binding, the chemical shift of the fully bound state must be determined. To achieve this, we carried out a global analysis of CSPs observed in the A_3_A_3_ and A_3_A_3_E_6_ RNC variants across multiple linker lengths (Fig. 4c). Two assumptions were required for this analysis. First, while the strength of interactions in these variants clearly varies as a function of linker length, we assume that the difference in free energy of binding between variants, ${\Delta}{\Delta}G_{{\mathrm{U}}_{{\mathrm{free}}} - {\mathrm{U}}_{{\mathrm{bound}}}}^{{\mathrm{A3A3}} - {\mathrm{A3A3E6}}}$ (where U_free_ and U_bound_ represent unfolded states with the C-terminal segment unbound and ribosome-bound, respectively), is independent of RNC length. This is equivalent to the assumption that C-terminal segments in both variants experience the same effective ribosome concentration at a given linker length, which is supported by the similar structural and dynamic properties of the A_3_A_3_ and A_3_A_3_E_6_ variants: no chemical shift perturbations or differences in ^15^N *R*_2_ relaxation rates are observed beyond the immediate vicinity of the E_6_ mutations (Extended Data Fig. 8). Second, while unbound resonance positions vary between A_3_A_3_ and A_3_A_3_E_6_ RNCs due to local sequence effects, we assume that the chemical shift change upon binding, Δ*δ*_max_, is the same for both variants. Given this, the observed chemical shift perturbations of four well-resolved resonances in A_3_A_3_ and A_3_A_3_E_6_ RNCs were fitted to determine the chemical shift differences between free and bound states, and the difference in affinities of the two variants, ${\Delta}{\Delta}G_{{\mathrm{U}}_{{\mathrm{free}}} - {\mathrm{U}}_{{\mathrm{bound}}}}^{{\mathrm{A3A3}} - {\mathrm{A3A3E6}}}= 1.9\pm0.1\,{\mathrm{kcal}}\,{{{\rm{mol}}^{-1}}}$ (Fig. 4d and Extended Data Fig. 8). These results indicate that at a short RNC length of 26 amino acids, 90% and 22% of the A_3_A_3_ and A_3_A_3_E_6_ RNCs are bound to the ribosome, respectively, then at a longer RNC length of 42 amino acids these values decreased to 60% and 7%, respectively, and by 110 amino acids, the interaction essentially disappears in the A_3_A_3_E_6_ variant (Fig. 4e).

**Extended Data Fig. 9:**
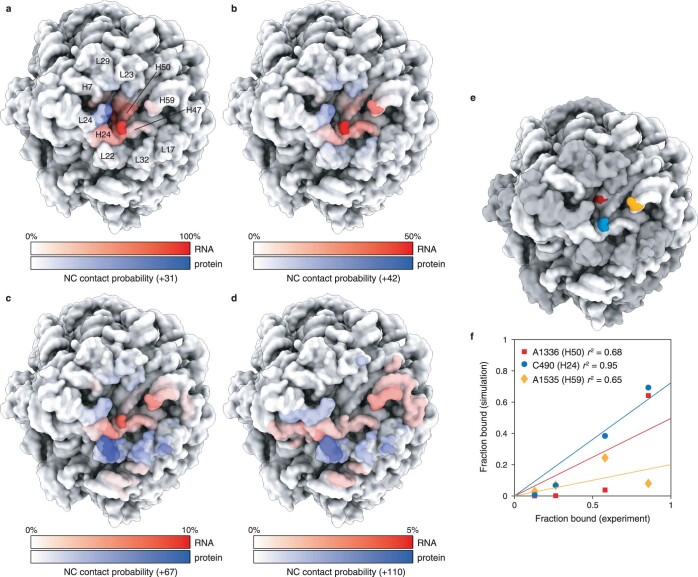
Molecular modelling of interactions between the FLN5 C-terminal region and the ribosome surface. (**a**–**d**) Contact probabilities between ribosome protein and RNA residues and the C-terminal region of FLN5 nascent chains, determined through coarse grained molecular dynamics simulations, for linker lengths (a) +31, (b) +42, (c) +67, and (d) +110. (**e**) Coarse-grained ribosome structure highlighting the location of RNA residues A1336, C490 and A1535, and (**f**) correlation plots between simulated and experimentally determined nascent chain–ribosome interactions for these residues. Data are shown for linker lengths +31, +42, +67 and +110, and best fit lines through the origin are plotted with correlation coefficients as indicated. Source data

Next, we used coarse-grained (CG) molecular dynamics simulations of the A_3_A_3_ and A_3_A_3_E_6_ RNCs to probe this interaction further, and to explore the location of nascent chain interaction sites on the ribosome surface (Fig. 4g). The strength of electrostatic interactions between the nascent chain and the ribosome surface in the CG model was calibrated using simulations of the A_3_A_3_ FLN5+42 RNC, in order to achieve ∼60% binding of the C-terminal segment (730–746), as determined from our NMR observations (Fig. 4e). Simulations of other RNC lengths (+31, +67 and +110), and of the +42 E_6_ variant, were then carried out with no further adjustment of parameters. We found that this CG model accurately identified electrostatics-based interactions of the C-terminal segment of the nascent chain that were consistent with the extent of binding measured from our CSP analysis across all lengths (Fig. 4f). The C-terminal binding segment (N728–C747) in the A_3_A_3_ FLN5+31 RNC contacted primarily the 23S rRNA region located adjacent to the exit tunnel (mainly helices H24 and H50), and also a loop of the nearby ribosomal protein, uL24 (Fig. 4h). Moreover, for many individual RNA bases in this region, in particular those within helices H24, H47, H50 and H59 (Extended Data Fig. 9), the contact probability correlated strongly with the extent of binding across nascent chain lengths as determined experimentally from our CSP analysis (Fig. 4i). These observations point to a nascent chain interaction site at the ribosome exit vestibule. We note that a similar interaction site is also observed for folded FLN5+47 RNC by cryo electron microscopy^28^, and that helices H24 and H50 are highly structurally conserved across Bacteria and Eukaryotes. It may be that this region on the ribosomal surface has a role in modulating the co-translational folding of FLN5 and perhaps generally for nascent chains.

### Modulation of co-translational folding by C-terminal interactions

**Fig. 5:**
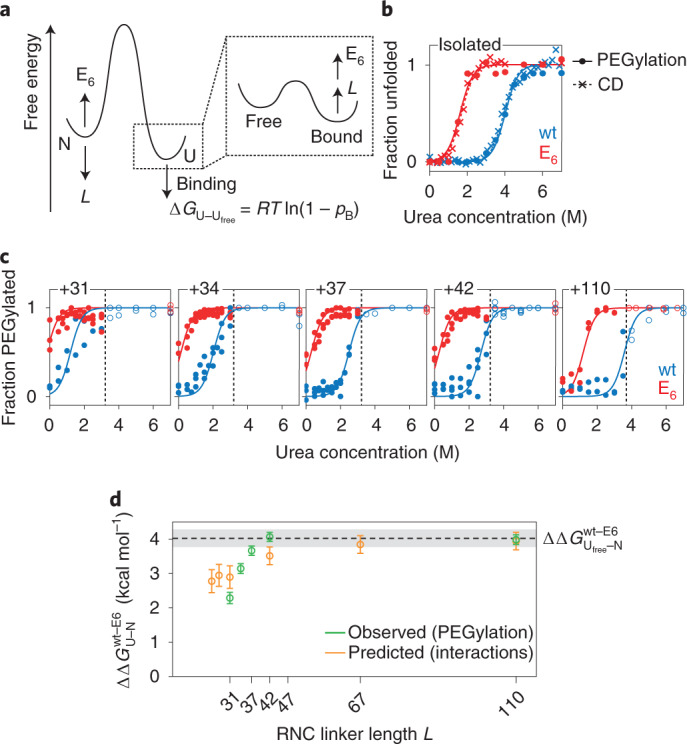
Analysis of folding and co-translational folding in FLN5 variants. **a**, Model for the free energy landscape associated with FLN5 RNCs, and how it is modulated by linker length *L*, and E_6_ mutations. **b**, Urea denaturation measurements of the stability of isolated wt and E_6_ FLN5 C721 V747, via the extent of PEGylation, and by CD spectroscopy. Solid lines show fits to a two-state unfolding model with a shared *m* value〈*m*_D–N_〉= 1.67 ± 0.06 kcal mol^−1^ M^−1^. **c**, Extent of PEGylation measured as a function of urea concentration for wt and E_6_ FLN5 RNCs. Measurements at low urea concentrations (filled circles) were fitted to a two-state unfolding curve using the *m* value determined from the isolated proteins. **d**, Difference in free energy of co-translational folding between wt and E_6_ RNCs, as measured by PEGylation (green) and predicted from CSP analysis (orange). Errors bars represent the standard error derived from fitting. Source data

In the previous sections, we have developed a detailed description of the interactions formed between unfolded FLN5 nascent chains and the ribosome surface. By stabilizing the unfolded state, such interactions will in general lead to an inhibition of folding (Fig. 5a), and we set out in this section to test this linkage in quantitative terms. Since interactions of the F_3_ and Y_3_ aromatic clusters are weak, we have focused our analysis on the strong interactions of the C-terminal segment, which we have previously shown is essential for folding of the domain^29^. A simple calculation (detailed in the Supplementary Information) relates the amount of binding within the unfolded state (*p*_B_) to its change in stability:

1 $$\Delta G_{{\rm{Ufree}}-{\rm{U}}} = RT \ln (1-p_{\rm{B}})$$

We have used this expression, together with our measurements of interactions in unfolded RNCs of various lengths, to predict the effect on the co-translational folding equilibria of folding-competent FLN5 RNCs in the absence of the destabilizing A_3_A_3_ mutations (Fig. 4e, right-hand axis). While this calculation assumes that the A_3_A_3_ mutations themselves do not perturb the interaction, we have previously noted that the impact of E_6_ mutations on interactions of wt and A_3_A_3_ RNCs was similar (Extended Data Fig. 7), and that there was no detectable cooperativity between the aromatic clusters and interactions of the C-terminal segment (Extended Data Fig. 5). On this basis, we predict a destabilization of wt FLN5 RNCs by over 1 kcal mol^−1^ at short linker lengths (Fig. 4e, right-hand axis). In the absence of other changes in thermodynamic stability, we also predict that the E_6_ variant should shift co-translational folding equilibria towards the folded state (Fig. 5a and Fig. 4e, right-hand axis).

**Extended Data Fig. 10:**
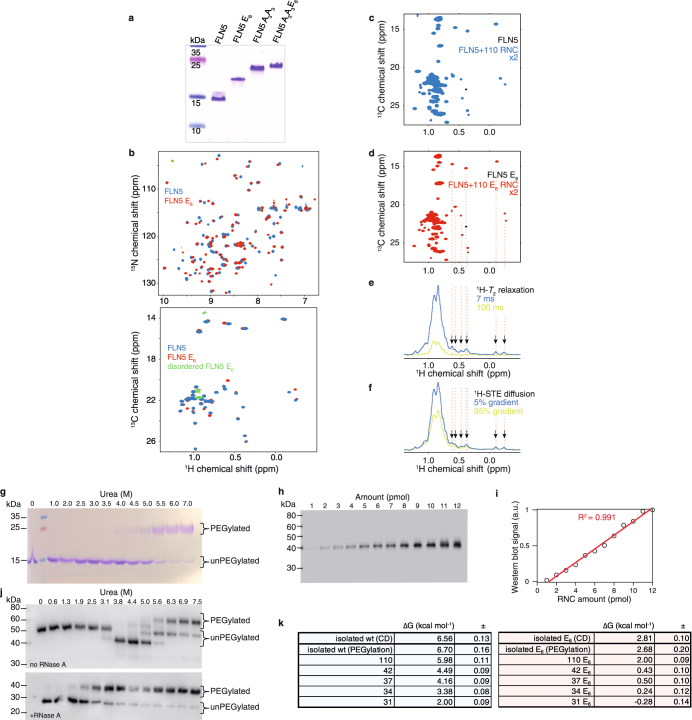
Folding of FLN5 and FLN5 E_6_ on and off the ribosome. (**a**) Coomassie-stained SDS PAGE gel of purified FLN5 protein variants. The net charge of FLN5 varies from -8.7 to -16.7 at pH 7.5 through the introduction of Glu residues, likely explaining the altered migration pattern on PAGE. (**b**) ^1^H,^15^N correlation spectra of ^15^N-labelled proteins (backbone), and ^1^H,^13^C correlation spectra of [^2^H,^13^CH_3_-ILV]-labelled proteins (sidechains). (**c**) ^1^H,^13^C HMQC spectrum of [^2^H,^13^CH_3_-ILV]-labelled FLN5+110 RNC. (**d**) Same as c, but for FLN5+110 E_6_ RNC. (**e**) Representative quality control measurements for nascent chain attachment by analysis of ^1^H transverse relaxation. Dispersed methyl resonances (indicated by arrows) detected after a 100 ms relaxation delay were taken as an indication of nascent chain release. (**f**) Representative quality control measurement by measurement of translational diffusion of dispersed nascent chain resonances (indicated by arrows) using ^1^H STE measurements with relative gradient strengths as indicated. (**g**) As a representative example, isolated FLN5 C721 V747 is shown on a Coomassie-stained 12% Bis-tris SDS-PAGE, after PEGylation measurements in urea. (**h**) As a representative example, FLN5+110 C721 V747 RNC is shown on a 12% Bis-tris SDS-PAGE, detected via anti-His tag western blot, after PEGylation measurements in urea. Intermediate concentrations of urea cause the tRNA-bound form of the nascent chain to migrate faster on Bis-Tris gels, as shown by the uniform migration pattern when the tRNA is degraded using RNase A, however this does not interfere with quantitation. (**i**) Western blot of 1-12 pmol of a FLN5+47 RNC. (**j**) Correlation plot of the western blot signal vs. concentration, from the blot shown in i. (**k**) Tabulated results from the fits of the data shown in Fig. 5b. The analysis was restricted to datapoints up to 3 M urea, and 3.75 M urea for FLN5+110 RNC^17^ (cf. solid markers in Fig. 5b). Source data

To test the predicted effect of interactions on folding experimentally, we employed a cysteine mass-tagging approach, PEGylation^30^, as a reporter of folding and co-translational folding equilibria. 2D NMR observations indicated that wt and E_6_ FLN5 have similar structures both in isolation and as RNCs (Extended Data Fig. 10), and so the mutations C747V and A721C were introduced to replace the native, C-terminal cysteine with one that is unambiguously emerged beyond the exit tunnel at all linker lengths examined here, and that is protected from modification by PEG-maleimide unless the domain is unfolded (Extended Data Fig. 10). The isolated variants, FLN5 C721 V747 (the ‘pseudo-wild-type’) and FLN5 C721 V747 E_6_, were reacted with PEG-maleimide in increasing concentrations of urea and the extent of protection was determined by electrophoresis. The resulting measurements fitted closely to a two-state unfolding model and indicated that the E_6_ variant was destabilized relative to the wt by ${\Delta}{\Delta}G_{{{{\rm{N}} - {\rm{U}}}}}^{{{{\rm{E6}} - {\rm{wt}}}}} = 4.0 \pm 0.2\,{\mathrm{kcal}}\,{{{\rm{mol}}^{-1}}}$ (where N and U represent native and unfolded states), in excellent agreement with equivalent CD measurements (Fig. 5b and Extended Data Fig. 10).

We next subjected FLN5 and FLN5 E_6_ RNCs to PEGylation following the same protocol (Fig. 5c and Extended Data Fig. 10). As ribosomes dissociate at high concentrations of urea^17^, we restricted our analysis to measurements below 3 M urea, with the exception of FLN5+110 RNC for which data up to 3.75 M were used (Fig. 5c, dashed lines). The stabilities of FLN5+110 wt and E_6_ RNCs are indistinguishable from the corresponding isolated proteins, indicating that the difference in thermodynamic stability between isolated FLN5 and FLN5 E_6_ is conserved on the ribosome at long linker lengths (${\Delta}{\Delta}G_{{{{\rm{N}} - {\rm{U}}}}}^{{{{\rm{E6}} - {\rm{wt}}}}}=4.0\pm0.3\,{\mathrm{kcal}}\,{{{\rm{mol}}^{-1}}}$). However, at short linker lengths, the difference in stability between FLN5 and FLN5 E_6_ was decreased (${\Delta}{\Delta}G_{{{{\rm{N}} - {\rm{U}}}}}^{{{{\rm{E6}} - {\rm{wt}}}}}=2.3\pm0.2\,{\mathrm{kcal}}\,{{{\rm{mol}}^{-1}}}$ for FLN5+31) (Fig. 5c,d). Consistent with our hypothesis, wt nascent chains therefore become destabilized relative to E_6_ under strongly interacting conditions. Based on our earlier measurements of bound populations (Fig. 4e), we can predict the change in stability relative to the isolated domains (Fig. 5d), and these predictions closely match direct observations using PEGylation (Fig. 5d). Therefore, we conclude that the C-terminal segment can indeed modulate co-translational FLN5 folding through the competition between binding and folding.

## Conclusion

For many domains, co-translational folding has been reported to be destabilized on the ribosome^5,17–19^. This effect is generally inferred to arise through interactions between emerging nascent chains and the ribosome, which have been observed for a range of different nascent chain sequences^12,21–24^. However, a direct link between the energetics of interactions and of co-translational folding has not previously been established. In this article we have therefore systematically examined a series of interactions between unfolded FLN5 nascent chains and the ribosome surface, in order to determine their effect on co-translational folding. While some of these interactions, between aromatic clusters and the ribosome surface, are too weak to perturb the energetics of folding substantially, we have identified a strongly interacting C-terminal segment, with over 90% bound at short linker lengths. The length of the sequestered segment (22 amino acids) is longer than the C-terminal truncation that can be tolerated by isolated FLN5 before it unfolds (12 amino acids)^29^, supporting the crucial role of this segment to enable native structure formation. Importantly, our analysis establishes quantitative agreement between the strength of the observed interactions and the energetics of co-translational folding itself, providing a residue-specific demonstration of the ability of the ribosome surface to directly modulate co-translational folding, effectively acting as a holdase.

A notable consequence of our analysis is that strong interactions are required to appreciably perturb the co-translational folding landscape, for example, a destabilization of 1 kcal mol^−1^ requires over 80% binding (equation (2)). Such interactions were indeed observed for the C-terminal segment at short linker lengths, but the bound population decreases sharply with increasing linker length, to below 50% at linker lengths beyond 47 amino acids (Fig. 4e). This rapid, short-range effect may provide a mechanism by which the engagement of molecular chaperones with the emerging NC is regulated.

We observe that the molecular determinants of ribosome interactions, that is, positively charged and aromatic residues, are similar to those recognized by other molecule chaperones and processing complexes^31^. These include signal recognition particle^32^, the ribosome-associated chaperone trigger factor^33–35^, and SecB^36^, both of which function as holdases for nascent polypeptide chains, and other downstream chaperones such as DnaK^37^. Indeed, over the past few years NMR studies have been instrumental in revealing with exquisite detail the mechanisms underlying substrate recognition and chaperone action by these molecules^35,36,38–42^.

During biosynthesis, substrates emerge from the ribosome in their high-energy unfolded states, and so in contrast to chaperones that act post-translationally, interactions with holdases carry no energetic cost. This is reflected in the ATP independence of both trigger factor and SecB holdases—as well as in interactions with the ribosome surface itself. We speculate that this short-range holdase activity could have a number of functional roles: sequestration of hydrophobic segments until later residues have been synthesized; to reduce the risk of misfolding, particularly in tandem repeat proteins such as FLN^29,43^; or to delay folding ahead of co-translational assembly^44–46^ or the engagement of downstream chaperones such as TF and SecB, which may be involved in secretory pathways^35,36^. This suggests that the ribosome is more than an inert hub that orchestrates interactions of auxiliary factors and chaperones^47^, and in fact is itself an active participant in the co-translational folding process. In conclusion, we demonstrate the holdase effect of the ribosome as a bespoke form of regulation over co-translational folding.

## Methods

### Sample preparation and quality control

DNA constructs of tandem immunoglobulin domains FLN5 and FLN6, and of poly(GS) glycine serine repeat sequence variants and Y719E variants, were described previously^20^. Aromatic and glutamate mutants were generated using site-directed mutagenesis. A stronger SecM variant was used for preparation of [^2^H,^13^CH_3_-Ile, Leu and Val (ILV)]-labelled RNCs for PEGylation experiments, with sequence ‘FSTPVWIWWWPRIRGPPPPWT’. ^15^N and [^2^H,^13^CH_3_-ILV]-labelled proteins were grown and purified as described previously^49^, except in the case of disordered FLN5 variants where the final chromatography step was performed using a HiLoad 16/600 Superdex 200 pg (GE Healthcare) column in the presence of 6 M urea, followed by buffer exchange into Tico buffer^25^. ^15^N and [^2^H,^13^CH_3_-ILV]-labelled RNCs were generated in BL21(DE3) *Escherichia coli* as described previously^25^, where the only modification was the replacement of the final step of purification (sucrose gradient chromatography) with an additional sucrose cushion step followed by hydrophobic affinity chromatography using a HiTrap FF butyl column (GE Healthcare). The occupancy and integrity of RNC samples were monitored as described previously^25^.

### NMR spectroscopy

NMR data were acquired on 500 and 700 MHz Bruker Avance III, and 800 and 950 MHz Bruker Avance III HD spectrometers, all equipped with TCI cryoprobes. Data were processed and analysed with nmrPipe, Sparky, CCPN Analysis and MATLAB^50,51^. All samples were prepared at nascent chain concentrations of between 7 and 13 µM in Tico buffer, pH 7.5, containing 10% D_2_O and 0.001% (w/v) DSS^25^. For ^15^N-labelled samples, ^1^H,^15^N SOFAST-HMQC experiments^52^ were recorded with typical acquisition times of 50.4 ms and 29.5 ms in the direct and indirect dimensions, respectively, and a 100 ms recycle delay. Transverse cross-correlated relaxation was measured using ^1^H,^15^N BEST-TROSY-CCR experiments (Extended Data Fig. 4), acquired at 950 MHz with a recycle delay of 200 ms and an acquisition time of 49 ms in the direct dimension. For measurements of isolated proteins, a relaxation delay of 111 ms was used, while shorter relaxation delays were used for measurements of RNCs (22 ms for FLN5+31; 44 ms for FLN5+31 F_3_A_3_ and FLN5+31 A_3_A_3_). An acquisition time of 43 ms was used in the indirect dimension for all measurements except for FLN5+31, for which a 21 ms acquisition time was used. Samples were doped with 15 mM NiDO2A (nickel(II) 1,4,7,10-tetraazacyclododecane-1,7-bis(acetic acid)) to enhance sensitivity (Extended Data Fig. 4)^53^. All experiments were interleaved with SORDID diffusion measurements^54^ with a diffusion delay of 190 ms, an acquisition time of 49 ms, and 4 ms trapezoidal gradient pulses with strengths of 0.058 and 0.387 T m^−1^. Ribosome background labelling was assessed using ^15^N filtered/edited difference spectroscopy^25^. Chemical shift assignments for FLN5 variants were verified using ^1^H,^15^N NOESY-HSQC and TOCSY-HSQC experiments, with 200 ms and 70 ms mixing times, respectively.

For [^2^H,^13^CH_3_-ILV]-labelled samples, ^1^H,^13^C HMQC spectra were acquired at 800 MHz with acquisition times of 100 ms and 7 ms in the direct and indirect dimensions, respectively. Sample integrity was assessed through ^13^C-edited ^1^H STE diffusion measurements^25^ and ^1^H *R*_2_ relaxation measurements (^13^C-edited and incorporating a filter to select slow relaxing coherences^55^), in which signals detected after a 100 ms relaxation delay were interpreted as indicating nascent chain release.

^1^H *R*_2_ rates of isolated amino acids (200 µM in D_2_O) were measured in the presence and absence of 1 µM 70S ribosomes, using a 500 Hz PROJECT pulse train (700 MHz, 283 K)^56^.

### CD spectroscopy

CD spectra were acquired using a Chirascan-plus CD spectrometer (Applied Photophysics). All samples (20 µM) were incubated for ≥3 h at 283 K prior to measurement in Tico buffer (12 mM Hepes, 30 mM NH_4_Cl, 6 mM MgCl_2_, pH 7.5). CD signals at 211 nm were fitted globally using MatLab to a two-state unfolding model with a common *m* value, 〈*m*_D–N_〉 (ref. ^57^):

2 $$\begin{array}{*{20}{c}} {y = \alpha _{\mathrm{N}} + \alpha _{\mathrm{D}}\frac{{e^{\frac{{\left\langle {m_{{\mathrm{D - N}}}} \right\rangle \left( {\left[ {\mathrm{D}} \right] - \left[ {\mathrm{D}} \right]_{50{{{\mathrm{\% }}}}}} \right)}}{{RT}}}}}{{1 + e^{\frac{{\left\langle {m_{{\mathrm{D - N}}}} \right\rangle \left( {\left[ {\mathrm{D}} \right] - \left[ {\mathrm{D}} \right]_{50{{{\mathrm{\% }}}}}} \right)}}{{RT}}}}}} \end{array}$$

where [D] is the denaturant concentration, [D]_50%_ is the midpoint of folding, and *α* terms represent baselines for native (N) and denatured states. Stabilities were then calculated as Δ*G*_D–N_ = *m*_D–N_[D]_50%_.

### Molecular dynamics

Simulations of FLN5+31, +42, +67 and +110 RNCs were run in Gromacs 4.5.7^58^ using Cα structure-based models generated with SMOG 2.0^59,60^, extended to represent RNA using three beads located at P, C4′ and N3 atoms. A rigid ribosome model was created based on the structure 4ybb^61^ and including only nascent chain-accessible residues, that is, the exit tunnel and the surrounding surface. The A_3_A_3_ variant was modelled by removing all contacts involving the mutated sites, and the simulation temperature was then tuned so that isolated wt and A_3_A_3_ variants were folded and unfolded, respectively. Electrostatic interactions were introduced using Debye–Hückel theory^62^, with parameters chosen to reproduce the experimentally observed bound population of the FLN5+42 RNC and then applied without change to the remaining lengths. Three independent simulations were carried out for each system, with ∼2 × 10^8^ steps per run.

### PEGylation

Samples (100–200 pmol isolated proteins or 8 pmol of RNC) were incubated in increasing urea concentrations for at least 2 h at 283 K prior to measurements. Methoxypolyethylene glycol maleimide (PEG-Mal) was added to a final concentration of 2 mM and incubated for 10 min before quenching the reaction with 0.23 M DTT. Samples were separated on denaturing 12% (w/v) polyacrylamide Bis-Tris gels (pH 5.7) which were subsequently Coomassie stained (for isolated proteins) or analysed by Western blot (for RNCs). Quantitation was performed using ImageStudio (Licor). As a control, Western blots of dilutions of a FLN5+47 RNC variant showed excellent linearity (*R*^2^ = 0.98) over the concentration range used in analyses (Supplementary Fig. 14). Populations of unfolded (PEGylated) nascent chains were determined relative to the total nascent chain concentration (PEGylated and unPEGylated) and fitted to equation (2) to determine the thermodynamic stability as described above.

## Online content

Any methods, additional references, Nature Research reporting summaries, source data, extended data, supplementary information, acknowledgements, peer review information; details of author contributions and competing interests; and statements of data and code availability are available at 10.1038/s41557-021-00796-x.

## Supplementary information

Supplementary Information Supplementary materials and methods.

## Data Availability

Data supporting the findings of this study are included in the article, source data and Supplementary Information files. Assignments have been deposited in the BMRB under accession codes 51023 and 51028. Source data are provided with this paper.

## Source data

Source Data Fig. 1 Source Data
Source Data Fig. 2 Source Data
Source Data Fig. 3 Source Data
Source Data Fig. 4 Source Data
Source Data Fig. 5 Source Data
Source Data Extended Data Fig. 1 Source Data
Source Data Extended Data Fig. 2 Source Data
Source Data Extended Data Fig. 2 Unprocessed gel data
Source Data Extended Data Fig. 3 Source Data
Source Data Extended Data Fig. 3 Unprocessed gel data
Source Data Extended Data Fig. 4 Source Data
Source Data Extended Data Fig. 4 Source code
Source Data Extended Data Fig. 5 Source Data
Source Data Extended Data Fig. 5 Unprocessed gel data
Source Data Extended Data Fig. 6 Source Data
Source Data Extended Data Fig. 7 Source Data
Source Data Extended Data Fig. 8 Source Data
Source Data Extended Data Fig. 9 Source Data
Source Data Extended Data Fig. 10 Source Data
Source Data Extended Data Fig. 10 Unprocessed gel data

## Extended data

are available for this paper at 10.1038/s41557-021-00796-x.

## Code availability

NMR pulse sequences are available on https://github.com/chriswaudby/pp and also as Supplementary Information. Source data are provided with this paper.
