# Supplementary material for: Interactions between nascent proteins and the ribosome surface inhibit co-translational folding
Source: Nat Chem. 2021 Oct 14;13(12):1214–20. doi: 10.1038/s41557-021-00796-x (PMC8627912; doi:10.1038/s41557-021-00796-x)
Supplement: Supplementary file 1 — Supplementary materials and methods. [file 41557_2021_796_MOESM1_ESM.pdf]

nature portfolio

https://doi.org/10.1038/s41557-021-00796-x

Supplementary information

Interactions between nascent proteins and the ribosome surface inhibit co-translational folding

In the format provided by the authors and unedited

SUPPLEMENTARY INFORMATION for

Interactions between nascent proteins and the ribosome surface inhibit co-translational folding

Anaïs M. E. Cassaignau[1,2], Tomasz Włodarski[1,2], Sammy H. S. Chan[1], Lauren F. Woodburn[1], Ivana V. Bukvin[1], Julian O. Streit[1], Lisa D. Cabrita[1], Christopher A. Waudby[1,*], John Christodoulou[1,2,*]

Materials and Methods

Analysis of nascent chain–ribosome interactions using transferred cross-correlated relaxation

The mobility of nascent chains on the ribosome is determined by a combination of segmental diffusion (similar to isolated disordered polypeptides), which may be restricted due to steric constraints around the ribosomal exit tunnel, and the interaction of the nascent chain with the ribosome surface to form a complex, motion within which is characterized by the rotational diffusion of the ribosome itself.

NMR measurements of nuclear spin relaxation provide a powerful route to the characterization of rotational diffusion and transferred relaxation due to transient interactions with larger macromolecular species can also provide a sensitive probe of weak molecular interactions[21]. In this work, we have focused on the measurement and analysis of transferred transverse cross-correlated relaxation (CCR) within amide spin systems. Cross-correlated relaxation originates as a difference in relaxation of a given nucleus depending on the spin-state of a neighboring nucleus (in this case, amide ^{15}N and ^{1}H nuclei respectively). A particular advantage of such processes is that they provide a 'pure' measurement of rotational diffusion, free from spin-state independent contributions such as chemical exchange, which may be significant for a protein close to its folding transition, or other external relaxation sources. This allows us to enhance substantially our experimental sensitivity by doping RNC samples with paramagnetic Ni(II) complexes, that accelerate transverse and particularly longitudinal proton

relaxation in a spin-state independent manner[53], without compromising the accuracy of our measurements.

The transverse cross-correlated relaxation rate, η_{xy}, for an amide ^{15}N spin is:

$$\eta_{xy} = \left(\frac{\mu_0 \hbar \gamma_H \gamma_N}{8\pi r_{NH}^3}\right)\left(\frac{\gamma_N B_0 \Delta\delta_N}{3}\right)[4J(0) + 3J(\omega_N)]P_2(\cos\theta)$$

where $P_2(x) = \frac{1}{2}(3x^2 - 1)$, $r_{\mathrm{NH}} = 1.02$ Å is the internuclear distance, $\theta \approx 17^{\circ}$ is the angle between the internuclear N–H vector and the principal axis of the ^{15}N chemical shift tensor, and $\Delta\delta_{\mathrm{N}} \approx 160$ ppm is the difference between the parallel and perpendicular components of the ^{15}N chemical shift tensor[63]. $J(\omega)$ is the spectral density associated with rotational diffusion, which for the ribosome is assumed to be isotropic with correlation time τ_{c}:

$$J(\omega) = \frac{2}{5} \cdot \frac{\tau_c}{1 + \omega^2 \tau_c^2}$$

The rotational correlation time of fluorescently labelled 70S ribosomes has previously been determined, using rotational depolarization of a long-lived triplet state, to be 3.3 μs cP^{-1}[27]. Under our experimental conditions (H_2O at 283 K), the solution viscosity is 1.3 cP and the ribosome rotational correlation time is therefore 4.3 μs. Therefore, the transverse CCR rate, η_{xy}, expected for a nascent chain residue in rigid association with the ribosome surface is approximately 7000 s^{-1} at the 22.3 T (950 MHz) field strength employed for these measurements in this work.

The CCR rate within the bound state may be reduced should this species have internal mobility, which may be characterized by an order parameter, $0 < S^2 \leq 1$, scaling the apparent CCR rate. In the limit of fast exchange, typical for weak interactions with a short residence time, the transferred CCR rate observed for a bound state population p_{B}, is therefore:

$$\Delta\eta_{xy} = S^2 p_B\left(\eta_{xy}^{\mathrm{bound}} - \eta_{xy}^{\mathrm{free}}\right) \approx S^2 p_B \eta_{xy}^{\mathrm{bound}}$$

Analysis of collinear chemical shift perturbations

Collinear chemical shift perturbations observed as a function of an external parameter are a hallmark of fast chemical exchange (on the chemical shift timescale) between two states. If the chemical shifts of both states are known, then the position of the equilibrium can be rapidly and accurately read out from such data. In the present case, we are considering exchange between free and ribosome-bound species, and so chemical shifts in the free state can be determined from spectra of isolated A_3A_3 or $A_3A_3E_6$ variants. The observed perturbation for a given nucleus, at a particular nascent chain linker length L, $\Delta\delta_{\text{obs}}(L)$, is therefore determined by the fraction bound, $p_B(L)$, together with the (unknown) chemical shift perturbation that would be expected in the fully bound state, $\Delta\delta_{\text{max}}$:

$$\Delta\delta_{\text{obs}}(L) = p_B(L)\Delta\delta_{\text{max}}$$

When $\Delta\delta_{\text{max}}$ is unknown, it is not possible to interpret CSPs quantitatively in terms of binding. However, by carrying out a joint analysis of CSPs at multiple lengths in two RNCs (in this case, A_3A_3 and $A_3A_3E_6$ variants), under mild assumptions it is possible to fully characterize the binding of both nascent chains (**Fig. 4b,c**).

The fraction bound may be related to the free energy of binding:

$$\Delta G_{Ubound-Ufree}(L) = -RT \ln\left(\frac{p_B(L)}{1 - p_B(L)}\right)$$

This interaction energy is dependent upon the nascent chain linker length, which modulates the effective concentration of the binding site on the ribosome surface. Provided that the E_6 mutations do not significantly perturb the (unfolded) structural ensemble of the A_3A_3 nascent chain, then we may reasonably assume that the free energy *difference* between two nascent chain variants, $\Delta\Delta G_{Ubound-Ufree}^{A3A3E6-A3A3} = \Delta G_{Ubound-Ufree}^{A3A3E6}(L) - \Delta G_{Ubound-Ufree}^{A3A3}(L)$, is independent of the nascent chain length. In addition, as chemical shifts are well described empirically by a series of

additive perturbations to the magnetic environment of the observed spins[64], we assume that the maximum chemical shift perturbation, $\Delta\delta_{\mathrm{max}}$, is equal for both variants. Experimentally, this assumption is supported by the observation that, while chemical shifts may be offset between variants due to the effects of the mutations, the relative angle of ^{1}H and ^{15}N shift perturbations is the same between variants **(Fig. 4b)**. We can therefore now relate CSPs observed at common lengths in both variants and fit these to determine $\Delta\delta_{\mathrm{max}}$ and $\Delta\Delta G_{Ubound-Ufree}^{A3A3E6-A3A3}$:

$$\Delta\delta_{A3A3E6}(L) = \frac{\Delta\delta_{A3A3}(L)\,\Delta\delta_{max}\exp\left(-\frac{\Delta\Delta G_{Ubound-Ufree}^{A3A3E6-A3A3}}{RT}\right)}{\Delta\delta_{A3A3}(L)\left[\exp\left(-\frac{\Delta\Delta G_{Ubound-Ufree}^{A3A3E6-A3A3}}{RT}\right)-1\right]+\Delta\delta_{max}}$$

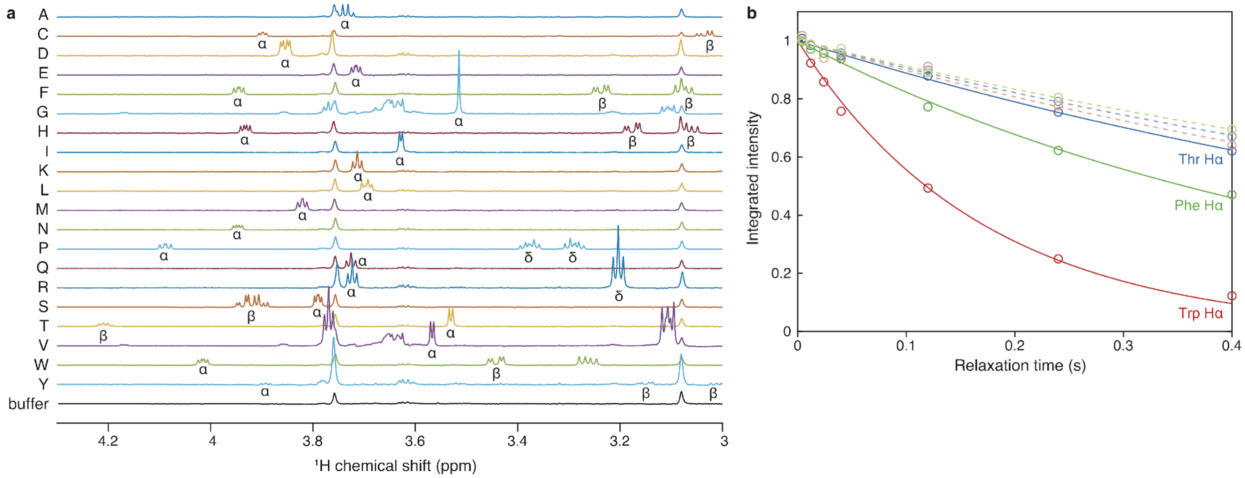

Fig. S1 | Interaction of free amino acid with empty 70S ribosomes. (a) Hα region of 1D ^{1}H NMR spectra of isolated amino acids (700 MHz, 283 K), showing atom assignments. (b) Representative ^{1}H R_2 relaxation measurements for the indicated amino acid resonances in the presence (solid lines) and absence (dashed lines) of 1 μM 70S ribosomes.

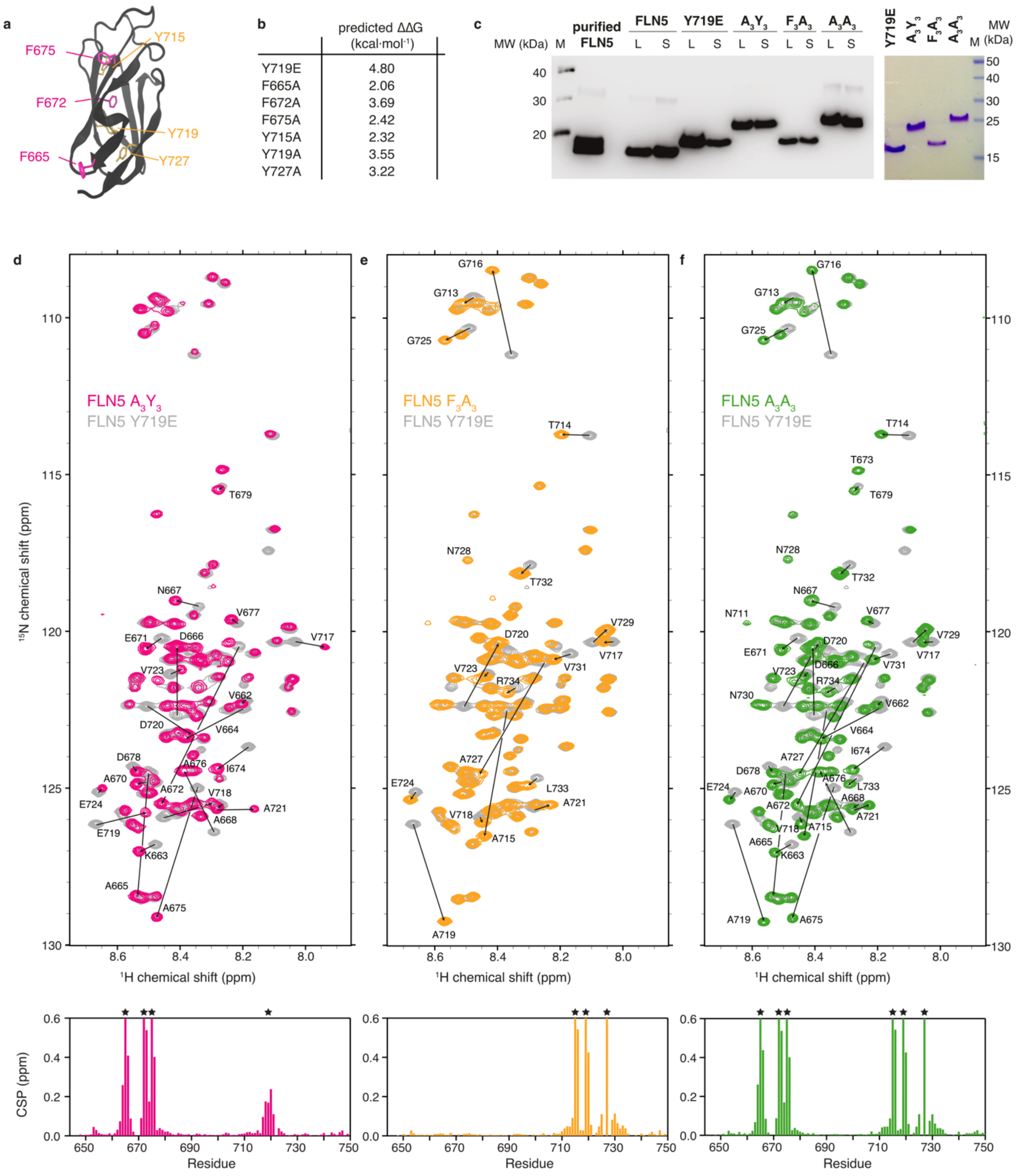

Fig. S2 | Biochemical and NMR analysis of isolated aromatic variants. (a) Aromatic residues that have been mutated in this study mapped onto the PDB structure of FLN5 (PDB 1QFH). (b) POPMUSIC stability predictions of the FLN5 aromatic mutations[65]. (c) Western blot (anti-His tag) of an expression text of FLN5, FLN5 Y719E, FLN5 A_3Y_3, FLN5 F_3A_3 and FLN5 A_3A_3 proteins (L: total lysate, S: soluble lysate) and Coomassie stained SDS PAGE gel of purified FLN5 Y719E, FLN5 A_3Y_3, FLN5 F_3A_3 and FLN5 A_3A_3 proteins. (d–f) ^{1}H,^{15}N correlation spectrum (283 K) of FLN5 Y719E overlaid with that of (d) FLN5 A_3Y_3, (e) FLN5 F_3A_3 and (f) FLN5 A_3A_3, with assignments of the shifted resonances indicated. Combined amide chemical

shift perturbations, $\Delta\delta = \sqrt{\Delta\delta_H^2 + (\Delta\delta_N/5)^2}$, are plotted below. The mutation sites relative to FLN5 Y719E have been indicated with star symbols on the CSP plot.

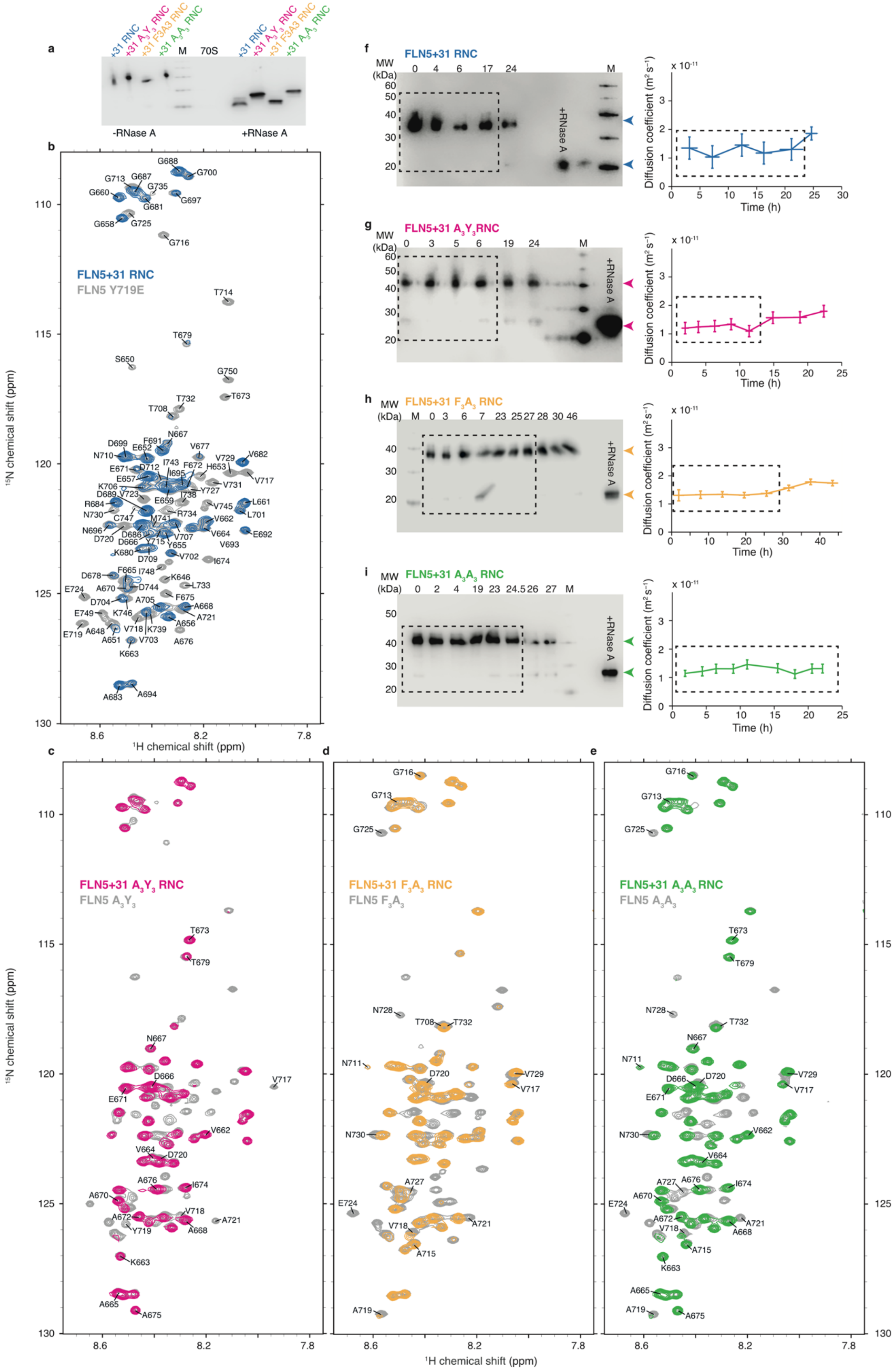
a
+31 RNC
+31 A3Y3 RNC
+31 F3A3 RNC
+31 A3A3 RNC
M
70S
-RNase A
+RNase A
b
FLN5+31 RNC
FLN5 Y719E
15N chemical shift (ppm)
1H chemical shift (ppm)
c
FLN5+31 A3Y3 RNC
FLN5 A3Y3
d
FLN5+31 F3A3 RNC
FLN5 F3A3
e
FLN5+31 A3A3 RNC
FLN5 A3A3
f
FLN5+31 RNC
MW (kDa)
+RNase A
Diffusion coefficient (m2 s-1)
x 10-11
Time (h)
g
FLN5+31 A3Y3RNC
h
FLN5+31 F3A3 RNC
i
FLN5+31 A3A3 RNC

Fig. S3 | Biochemical and NMR analysis of aromatic RNC variants. (a) Anti-His tag western blot of FLN5+31 wt, A_3Y_3, F_3A_3 and A_3A_3 RNCs (10 pmol each) purified for NMR studies, in their tRNA-bound and released forms. The migration patterns on SDS page reflect those of the isolated mutant proteins (cf. Supplementary Fig. 2). (b–e) ^{1}H,^{15}N SOFAST-HMQC spectra (283 K, 950 MHz) of (b) FLN5+31, (c) FLN5+31 A_3Y_3, (d) FLN5+31 F_3A_3 and (e) FLN5+31 A_3A_3 RNCs against the corresponding isolated, unfolded FLN5 variants, with assignments. (f–i) Assessment of the integrity of ^{15}N-labelled RNCs[25]: Anti-His western blots and translational diffusion coefficients measured using ^{15}N SORDID experiments are shown. The timeframes during which nascent chains have been assessed as being intact and used for analysis are indicated (dashed boxes). Vertical error bars represent standard errors derived from the spectral noise; horizontal bars (where applicable) indicate the acquisition periods of diffusion measurements.

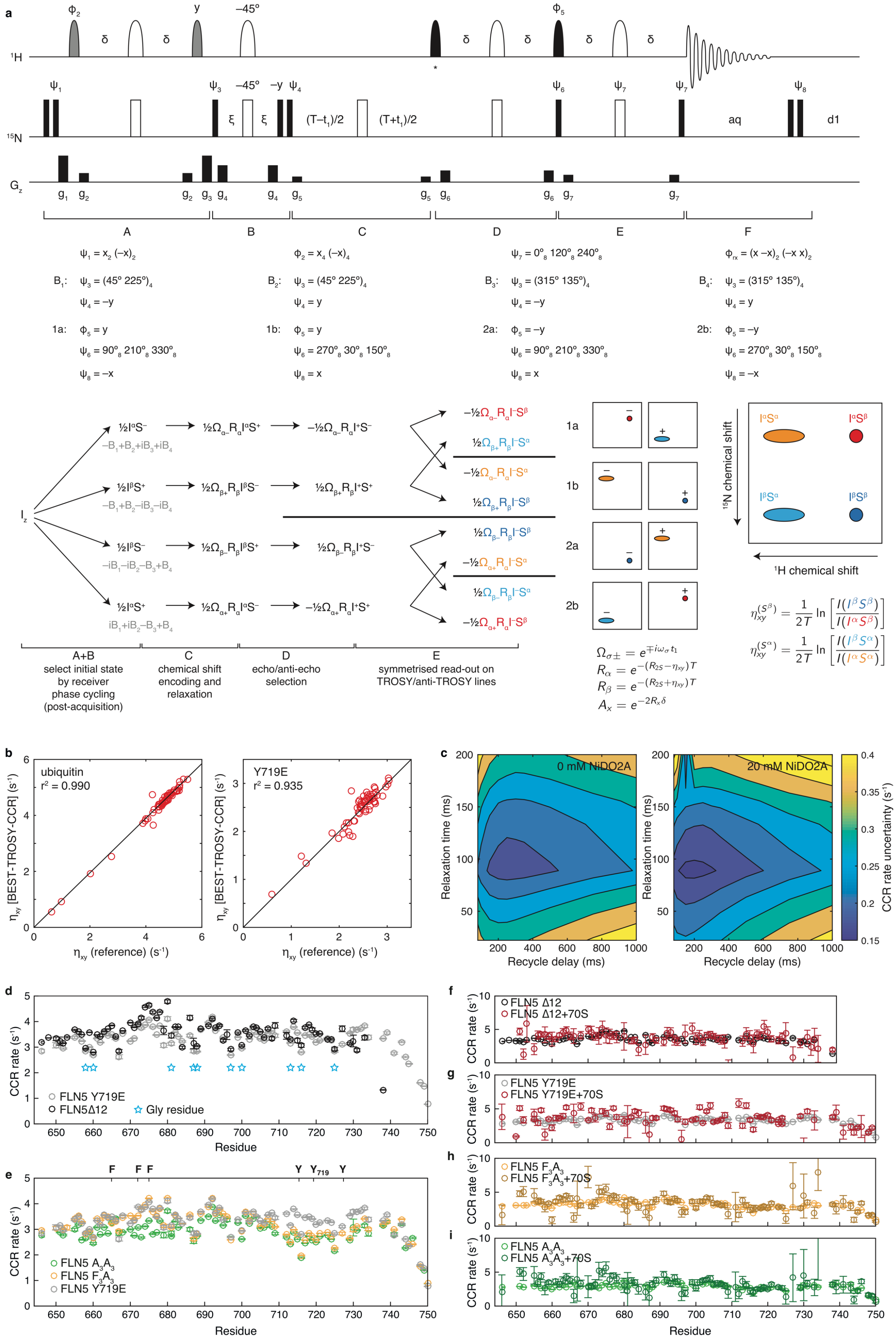
a
^{1}H
^{15}N
G_z
ϕ2 δ δ y −45° ϕ5 δ δ δ δ
ψ1 ψ3 −45° −y ψ4 ξ ξ (T−t1)/2 (T+t1)/2 ψ6 ψ7 ψ7 aq ψ8 d1
g1 g2 g2 g3 g4 g4 g5 g5 g6 g6 g7 g7
A B C D E F
ψ1 = x2 (−x)2
ϕ2 = x4 (−x)4
ψ7 = 0°8 120°8 240°8
ϕrx = (x −x)2 (−x x)2
B1: ψ3 = (45° 225°)4 ψ4 = −y
B2: ψ3 = (45° 225°)4 ψ4 = y
B3: ψ3 = (315° 135°)4 ψ4 = −y
B4: ψ3 = (315° 135°)4 ψ4 = y
1a: ϕ5 = y ψ6 = 90°8 210°8 330°8 ψ8 = −x
1b: ϕ5 = y ψ6 = 270°8 30°8 150°8 ψ8 = x
2a: ϕ5 = −y ψ6 = 90°8 210°8 330°8 ψ8 = x
2b: ϕ5 = −y ψ6 = 270°8 30°8 150°8 ψ8 = −x
A+B select initial state by receiver phase cycling (post-acquisition)
C chemical shift encoding and relaxation
D echo/anti-echo selection
E symmetrised read-out on TROSY/anti-TROSY lines
1a 1b 2a 2b
^{15}N chemical shift
^{1}H chemical shift
b
ubiquitin r² = 0.990
Y719E r² = 0.935
ηxy [BEST-TROSY-CCR] (s⁻¹)
ηxy (reference) (s⁻¹)
c
0 mM NiDO2A
20 mM NiDO2A
Relaxation time (ms)
Recycle delay (ms)
CCR rate uncertainty (s⁻¹)
d
FLN5 Y719E
FLN5Δ12
Gly residue
CCR rate (s⁻¹)
Residue
e
F F F Y Y719 Y
FLN5 A3A3
FLN5 F3A3
FLN5 Y719E
f
FLN5 Δ12
FLN5 Δ12+70S
g
FLN5 Y719E
FLN5 Y719E+70S
h
FLN5 F3A3
FLN5 F3A3+70S
i
FLN5 A3A3
FLN5 A3A3+70S

Fig. S4 | The BEST-TROSY-CCR experiment and application to isolated FLN5 variants. (a) Pulse sequence and processing scheme for the BEST-TROSY-CCR experiment. Solid coloring indicates 90º pulses; empty shapes indicate 180º pulses. ^{1}H pulse lengths are calculated for application at an offset of 8.2 ppm (950 MHz). Gray ^{1}H 90º pulses are Pc9_4_90 pulses (1958 µs), solid ^{1}H 90º shapes indicate Eburp2 pulses (1251 µs, * indicates time reversed pulse), and ^{1}H 180º shapes indicate Reburp pulses (1432 µs). ^{15}N pulses are applied as high-power rectangular pulses. 16 subspectra are acquired, corresponding to the possible combinations of the phase programs B_1–B_4 and the phase programs 1a,1b,2a and 2b as indicated. All pulses are applied with a phase of x (0º) unless otherwise indicated. ^{15}N phases are shown as spin dynamical phases and must be modified for application on Bruker spectrometers[66]. Gradients are applied as SMSQ shapes: g_1 (31.4%, 1 ms), g_2 (23%, 300 µs), g_3 (21%, 1 ms), g_4 (31%, 300 µs), g_5 (11%, 1 ms), g_6 (16.7%, 300 µs), g_7 (45%, 300 µs). (b) Comparison of CCR rates measured for ubiquitin and FLN5 Y719E using our pulse sequence (111 ms relaxation delay, 283K) against a reference measurement using symmetric recombination[67]. (c) Sensitivity enhancement on the CCR measurements of FLN5 Y719E (283 K, 950 MHz) obtained using the PLRE agent NiDO2A, an inert Ni^{II} chelate. (d) CCR rates of FLN5 Y719E and FLN5Δ12. Blue stars indicate Gly residues, which on average have lower CCR rates. (e) CCR rates of FLN5 Y719E, FLN5 F_3A_3 and FLN5 A_3A_3. (f–i) Comparison of CCR rates of FLN5 variants at 10 µM, in presence and absence of an equimolar concentration of 70S ribosomes: (f) FLN5Δ12, (g) FLN5 Y719E, (h) FLN5 F_3A_3 and (i) FLN5 A_3A_3. All errors were derived from the spectral noise.

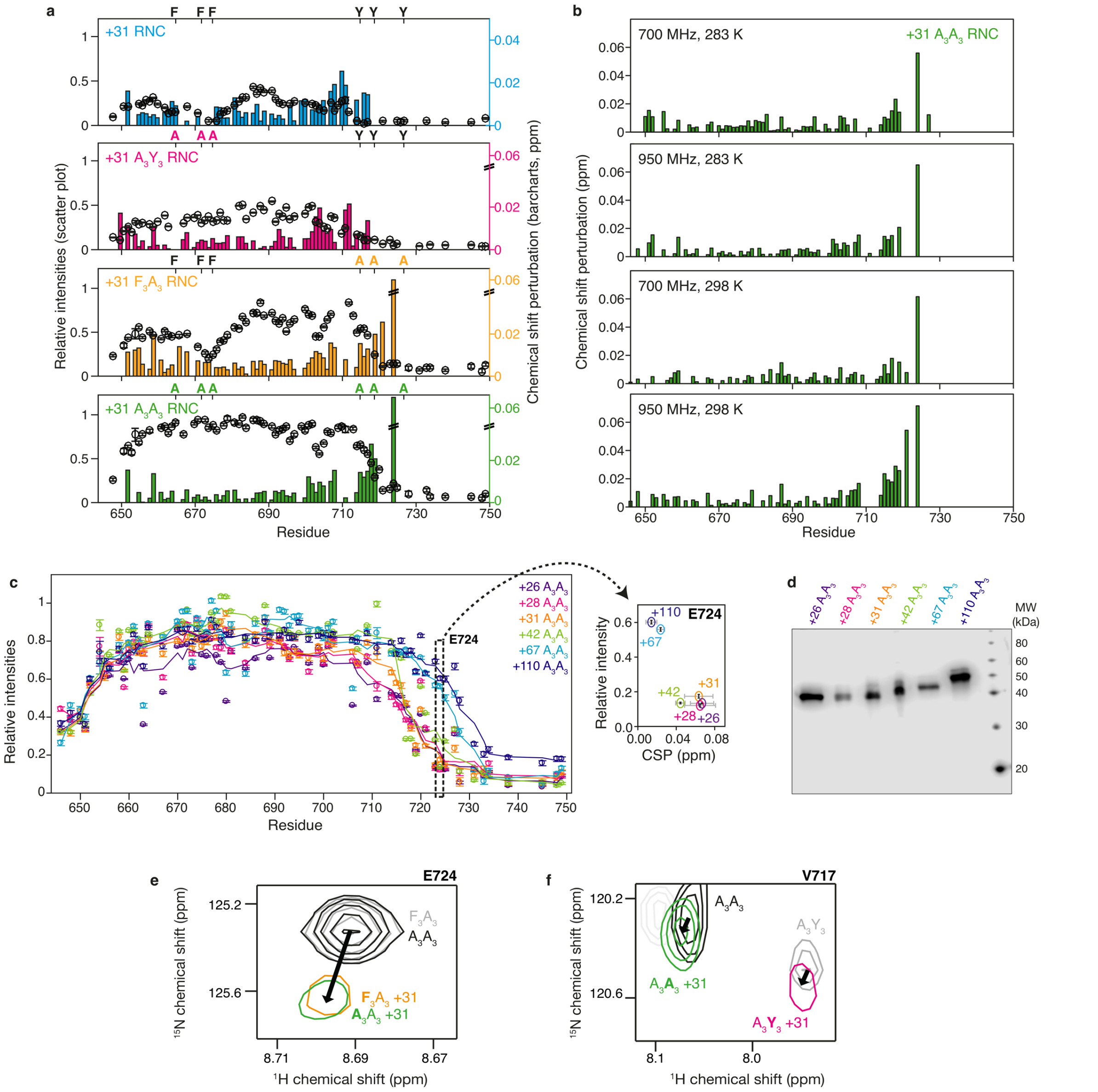

Fig. S5 | CSPs of FLN5 RNCs against isolated FLN5 variants, and comparison of chemical shift perturbations in the C-terminal region of FLN5 RNCs between aromatic cluster variants. (a) Relative intensities (LH axis) of FLN5+31, FLN5+31 A_3Y_3, FLN5+31 F_3A_3 and FLN5+31 A_3A_3 RNCs (283 K) and chemical shift perturbations (RH axis, $\Delta\delta = \sqrt{\Delta\delta_H^2 + (\Delta\delta_N/5)^2}$)), relative to their corresponding isolated, unfolded FLN5 variants. (b) Chemical shift perturbations of FLN5+31 A_3A_3 RNC relative to isolated FLN5 A_3A_3, at fields and temperatures as indicated. (c) Relative intensities of FLN5+26, 28, 31, 42, 67, 110 A_3A_3 RNCs relative to isolated FLN5 A_3A_3 (283 K, 950 MHz). inset: Intensities and combined amide chemical shift perturbations ($\Delta\delta = [\Delta\delta_H^2 + (\Delta\delta_N/5)^2]^{1/2}$) of the E724 amide resonance observed relative to isolated FLN5 A_3A_3 in ^{1}H,^{15}N SOFAST-HMQC spectra of FLN5 A_3A_3

RNCs, with varying linker lengths as indicated. (d) Anti-His tag western blot of FLN5+26, 28, 31, 42, 67, 110 A_3A_3 RNCs purified for NMR studies, in their tRNA-bound form.

(e) Comparison of C-terminal chemical shift perturbations between A_3A_3 and F_3A_3 variants. The difference in perturbations between A_3A_3 and F_3A_3 variants was 0.009 ± 0.050 ppm (^{15}N) and 0.002 ± 0.006 ppm (^{1}H). (f) Comparison of C-terminal chemical shift perturbations between A_3A_3 and A_3Y_3 variants. Due to chemical shift perturbations between the A_3A_3 and A_3Y_3 variants, and the reduced intensity of C-terminal residues in the A_3Y_3 variant **(Fig. 2b)**, E724 cannot be resolved in the A_3Y_3 spectrum and residue V717 is analyzed instead. The difference in perturbations between A_3A_3 and A_3Y_3 variants was 0.060 ± 0.044 ppm (^{15}N) and 0.001 ± 0.006 ppm (^{1}H).

Based on these negligible differences, mutations in the F_3 and Y_3 clusters do not seem to affect the CSP which report on the ribosome interaction of the C-terminal segment (residues N728 to C747).

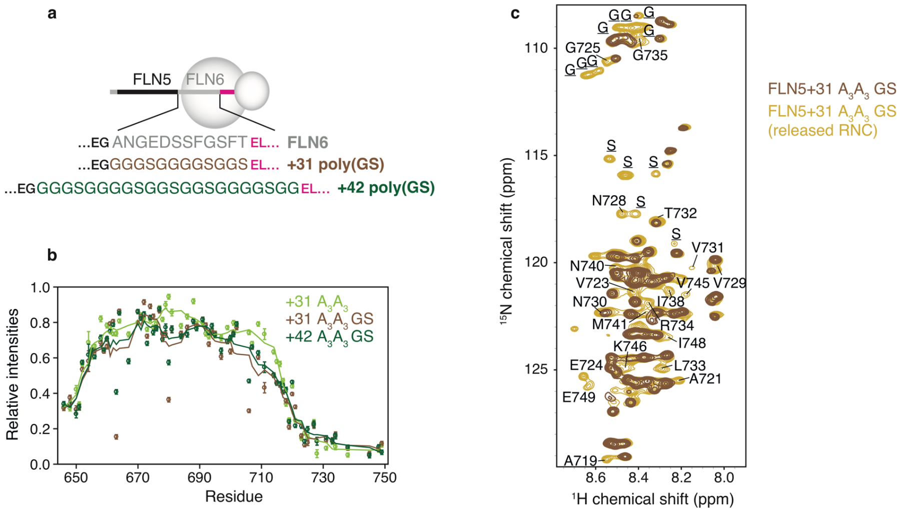

Fig. S6 | Analysis of FLN5-poly(GS) RNCs. (a) Design of GS linker RNCs. (b) ^{1}H,^{15}N SOFAST-HMQC resonances intensities for FLN5+31 A_3A_3, +31 A_3A_3 GS and +42 A_3A_3 GS RNCs relative to isolated FLN5 A_3A_3. (c) Overlay of a ^{1}H,^{15}N spectrum of FLN5+31 A_3A_3 GS RNC and a ^{1}H,^{15}N spectrum of the same RNC after treatment with 20 mM EDTA to induce nascent chain release. The residues only observable in EDTA-treated FLN5+31 A_3A_3 GS RNC are assigned, including resonances marked “G” and “S” which we attribute to the poly(GS) linker.

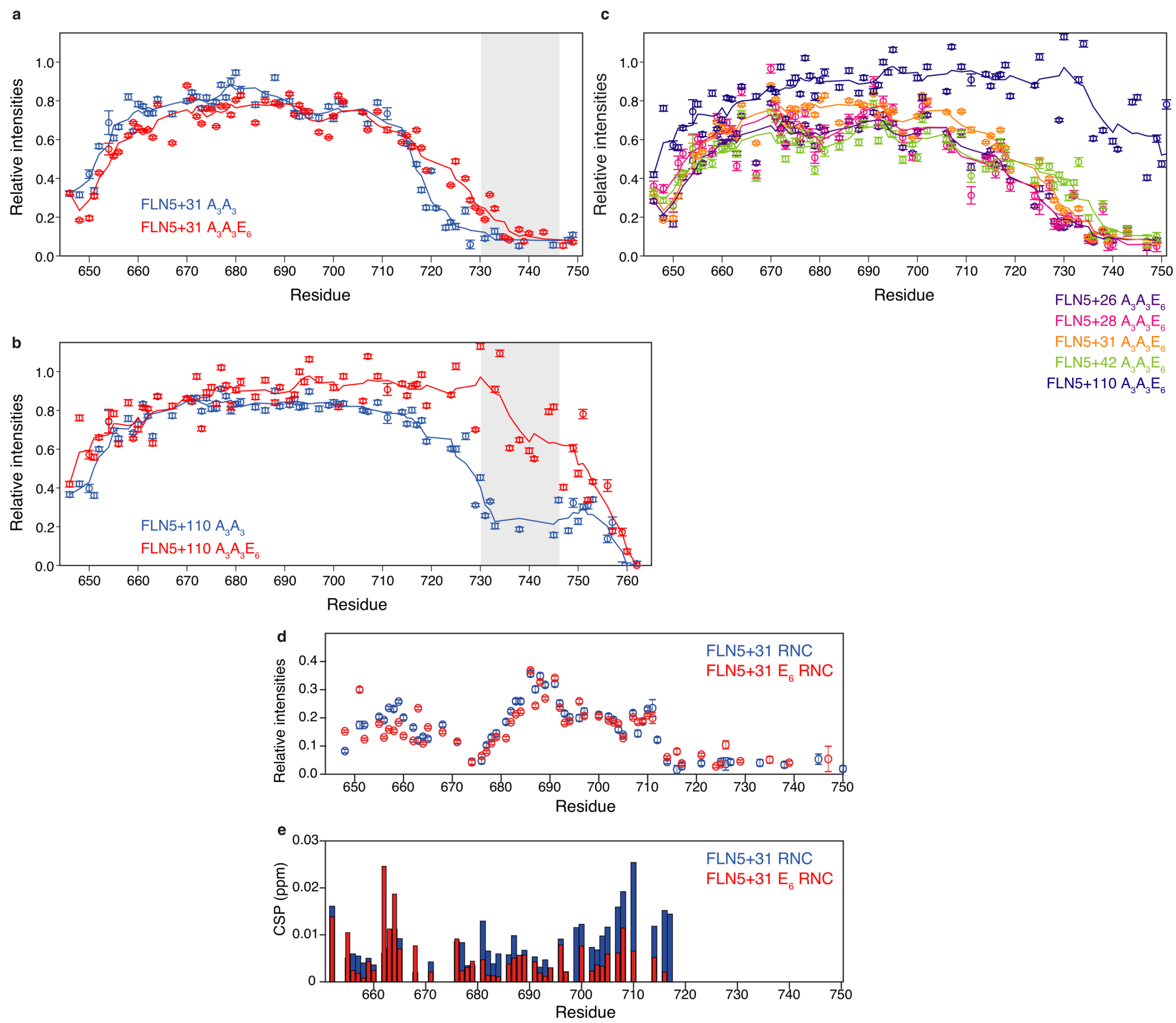

Fig. S7 | Characterization of FLN5 E_6 variants. (a) ^{1}H,^{15}N SOFAST-HMQC resonance intensities of FLN5+31 A_3A_3 RNC relative to isolated FLN5 A_3A_3, and of FLN5+31 $A_3A_3E_6$ RNC relative to isolated FLN5 $A_3A_3E_6$ (283 K, 950 MHz). The region between residues 730-746 is highlighted in light grey. (b) Same as in (a), except for FLN5+110 A_3A_3 and $A_3A_3E_6$ RNCs. (c) ^{1}H,^{15}N SOFAST-HMQC resonance intensities of FLN5+26, 28, 31, 42, 110 A_3A_3 E_6 RNCs relative to isolated FLN5 $A_3A_3E_6$ (283 K, 950 MHz). All errors were derived from the spectral noise. (d) Relative intensities of FLN5+31 and FLN5+31 E_6 RNCs (283 K) relative to their corresponding isolated, unfolded FLN5 variants. (e) Chemical shift perturbations ($\Delta\delta = \sqrt{\Delta\delta_H^2 + (\Delta\delta_N/5)^2}$) of FLN5+31 and FLN5+31 E_6 RNCs (283 K) relative to their corresponding isolated, unfolded FLN5 variants.

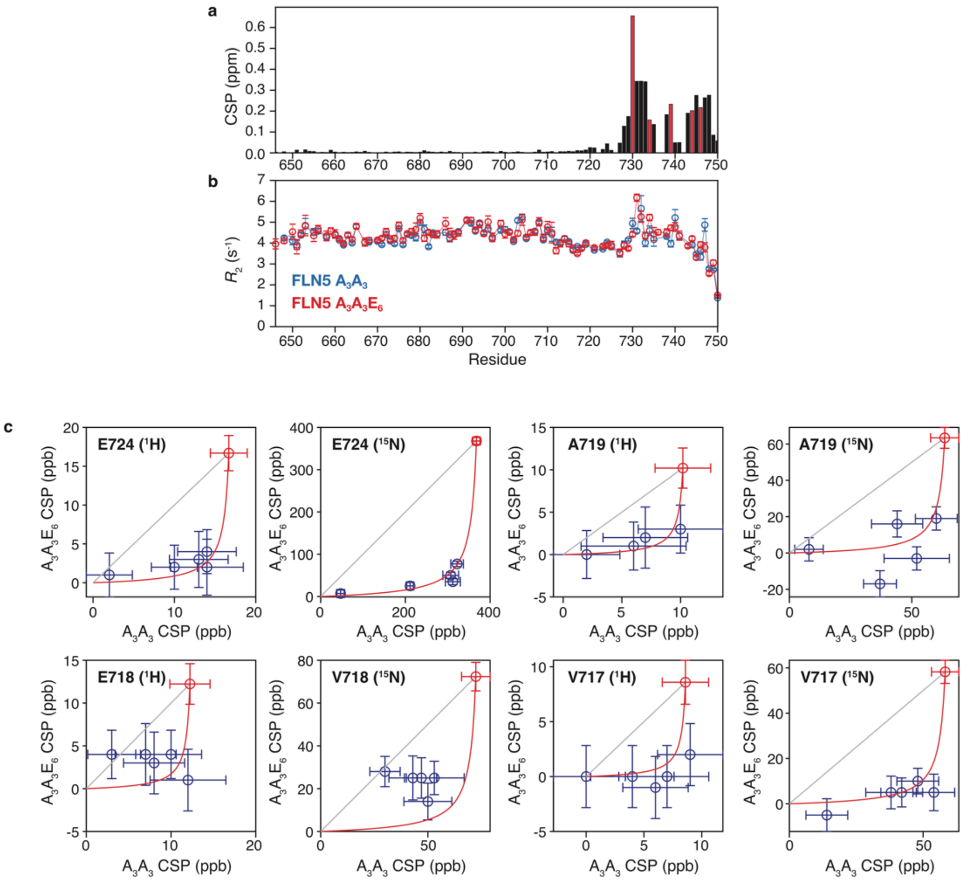

Fig. S8 | Global analysis of FLN5 A_3A_3 and $A_3A_3E_6$ chemical shift perturbations. (a) Chemical shift perturbations of FLN5 A_3A_3 *vs.* FLN5 $A_3A_3E_6$ ($\Delta\delta = \sqrt{\Delta\delta_H^2 + (\Delta\delta_N/5)^2}$). E_6 mutation sides are indicated using red bar charts. (b) T_2 relaxation measurements of isolated FLN5 A_3A_3 and FLN5 $A_3A_3E_6$. (c) Correlation plots of ^{1}H and ^{15}N chemical shift perturbations in +26, +28, +31, +42 and +110 A_3A_3/$A_3A_3E_6$ RNCs for residues as indicated (283 K, 950 MHz). A global fit of $\Delta\Delta G_{Ubound-Ufree}^{A3A3-A3A3E6}$ is shown (red line) and red markers indicate the fitted values of $\Delta\delta_{max}$ (χ^2 = 70.17, dof = 66, χ^2/ν = 1.06).

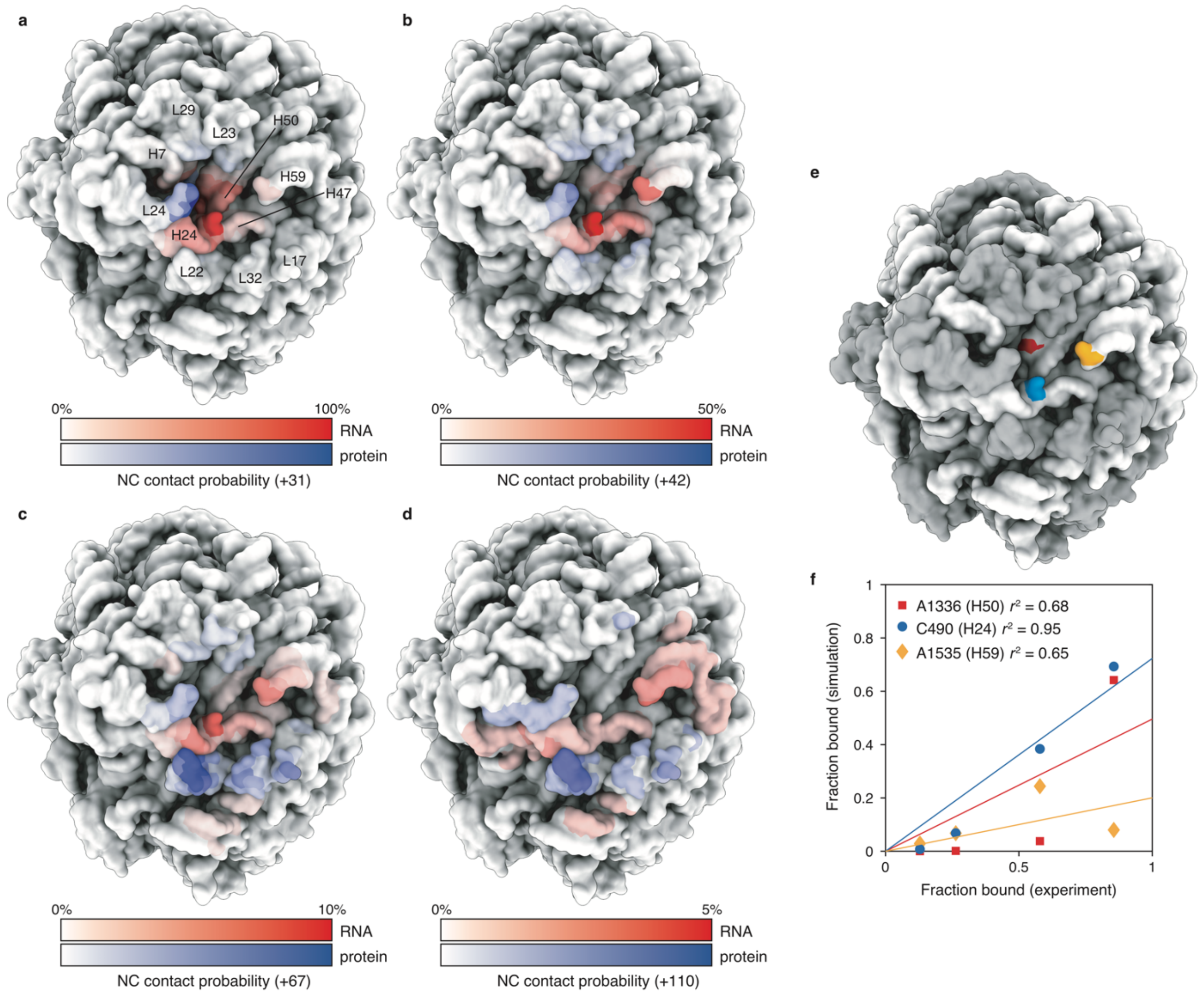

Fig. S9 | Molecular modelling of interactions between the FLN5 C-terminal region and the ribosome surface. (a–d) Contact probabilities between ribosome protein and RNA residues and the C-terminal region of FLN5 nascent chains, determined through coarse grained molecular dynamics simulations, for linker lengths (a) +31, (b) +42, (c) +67, and (d) +110. (e) Coarse-grained ribosome structure highlighting the location of RNA residues A1336, C490 and A1535, and (f) correlation plots between simulated and experimentally determined nascent chain–ribosome interactions for these residues. Data are shown for linker lengths +31, +42, +67 and +110, and best fit lines through the origin are plotted with correlation coefficients as indicated.

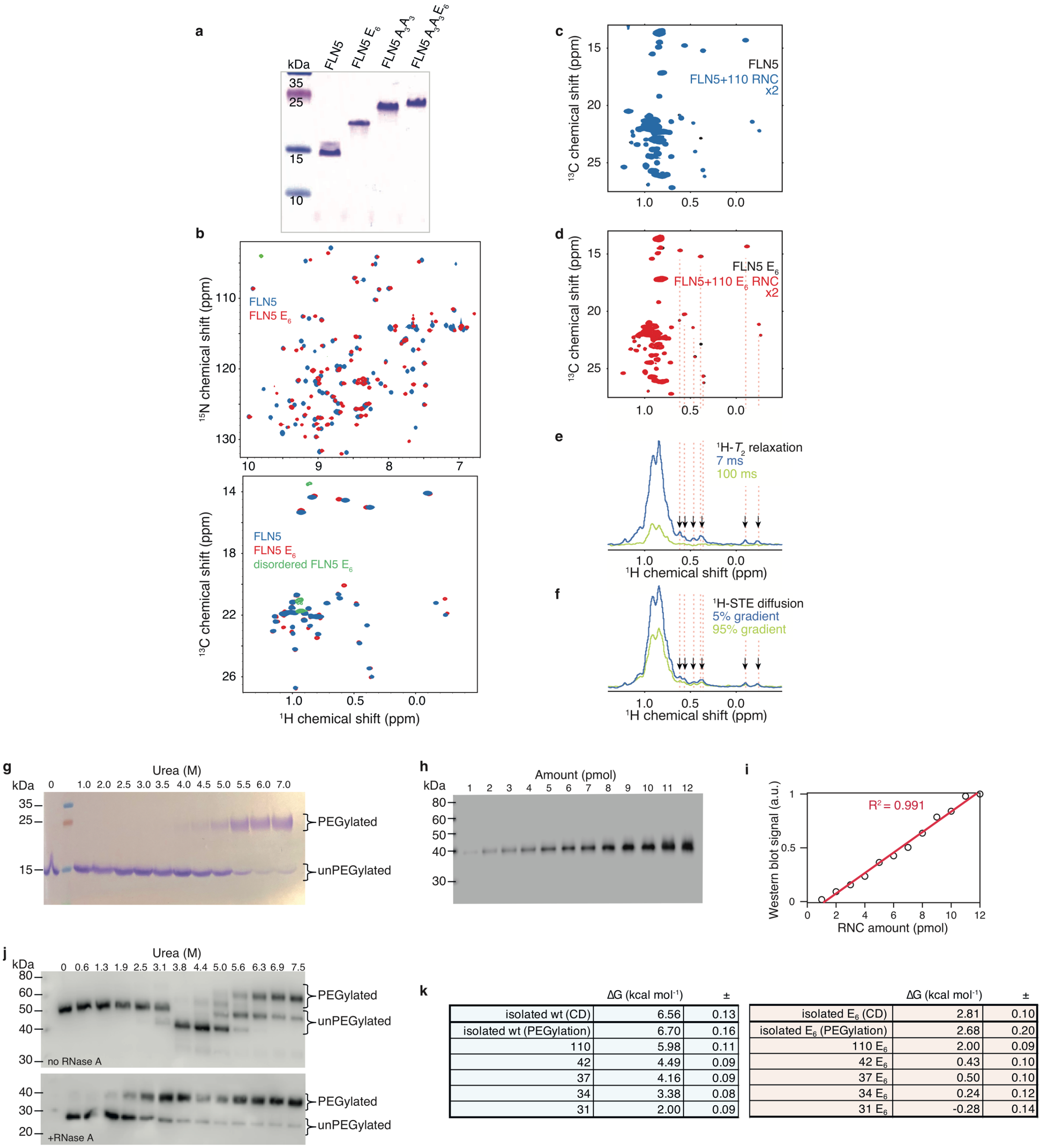

	ΔG (kcal mol-1)	±
isolated wt (CD)	6.56	0.13
isolated wt (PEGylation)	6.70	0.16
110	5.98	0.11
42	4.49	0.09
37	4.16	0.09
34	3.38	0.08
31	2.00	0.09

	ΔG (kcal mol-1)	±
isolated E_6 (CD)	2.81	0.10
isolated E_6 (PEGylation)	2.68	0.20
110 E_6	2.00	0.09
42 E_6	0.43	0.10
37 E_6	0.50	0.10
34 E_6	0.24	0.12
31 E_6	-0.28	0.14

Fig. S10 | Folding of FLN5 and FLN5 E_6 *on* and *off* the ribosome.
(a) Coomassie-stained SDS PAGE gel of purified FLN5 protein variants. The net charge of FLN5 varies from -8.7 to -16.7 at pH 7.5 through the introduction of Glu residues, likely explaining the altered migration pattern on PAGE. (b) ^{1}H,^{15}N correlation spectra of ^{15}N-labelled proteins (backbone), and ^{1}H,^{13}C correlation spectra of [^{2}H,$^{13}CH_3$-ILV]-labelled proteins (sidechains). (c) ^{1}H,^{13}C HMQC spectrum of [^{2}H,$^{13}CH_3$-ILV]-labelled FLN5+110 RNC. (d) Same as c, but for FLN5+110 E_6 RNC. (e) Representative quality control measurements for nascent chain attachment by analysis of ^{1}H transverse relaxation. Dispersed methyl resonances (indicated by arrows) detected after a 100 ms relaxation delay were taken as an indication of nascent chain release. (f) Representative quality control measurement by measurement of translational diffusion of dispersed nascent chain resonances (indicated by arrows) using ^{1}H STE

measurements with relative gradient strengths as indicated. (g) As a representative example, isolated FLN5 C721 V747 is shown on a Coomassie-stained 12% Bis-tris SDS-PAGE, after PEGylation measurements in urea. (h) As a representative example, FLN5+110 C721 V747 RNC is shown on a 12% Bis-tris SDS-PAGE, detected via anti-His tag western blot, after PEGylation measurements in urea. Intermediate concentrations of urea cause the tRNA-bound form of the nascent chain to migrate faster on Bis-Tris gels, as shown by the uniform migration pattern when the tRNA is degraded using RNase A, however this does not interfere with quantitation. (i) Western blot of 1-12 pmol of a FLN5+47 RNC. (j) Correlation plot of the western blot signal vs. concentration, from the blot shown in i. (k) Tabulated results from the fits of the data shown in Fig 5b. The analysis was restricted to datapoints up to 3 M urea, and 3.75 M urea for FLN5+110 RNC[17] (cf. solid markers in Fig 5b).

Description of the BEST-TROSY-CCR experiment

A variety of methods have been described for the measurement of the transverse cross-correlated relaxation rate η_{xy}[63,67-70]. We have built upon these to produce a new experiment, BEST-TROSY-CCR, with optimized sensitivity **(Supplementary Fig. 4)**. This experiment combines a spin-state selective excitation (S^3E) element[69], sensitivity enhancement and longitudinal relaxation optimization[70] in a constant time *J*-resolved experiment[68] in which collection and post-processing of a series of sub-spectra allows as far as possible the simultaneous acquisition and isolation of all magnetization transfer pathways and multiplet components.

The experiment is designed to prepare and observe the chemical shift evolution of $I^\alpha S^\pm$ and $I^\beta S^\pm$ coherences during the constant time period C **(Supplementary Fig. 4a)**, of total length $T = n/J_{NH}$. Relaxation of these coherences occurs in a spin-state dependent manner, with rates $R_{2\beta} = R_{2S} + \eta_{xy}$ and $R_{2\alpha} = R_{2S} - \eta_{xy}$. This constant time evolution period has two advantages: first, chemical shift evolution and relaxation are simultaneous, resulting in a shorter overall sequence and improved sensitivity; and secondly, the experiment is robust to proton spin flips occurring during this period, which are strongly suppressed due to their non-secular nature. The resulting magnetization is then read out with spin-state selective detection. Critically, coherences are prepared and observed in a way that ensures all relaxation processes affect spin states equally[67]. Magnetization can be read out using both TROSY ($I^- S^\beta$) and anti-TROSY ($I^- S^\alpha$) components of the proton doublet, providing two independent measurements that can be combined for additional sensitivity. From the observed intensities of the α and β transitions of the nitrogen doublet, the cross-correlated transverse relaxation rate, η_{xy}, may then be calculated.

The experiment is acquired as a series of 16 subspectra, corresponding to all possible combinations of the phase programs B_1–B_4 and 1a, 1b, 2a and 2b as shown in Supplementary Fig. 4a. These can be analyzed most effectively by evaluating the evolution of magnetization through the individual sections of the pulse sequence, A–F. From this analysis, linear combinations of the acquired subspectra can then be derived and calculated through post-acquisition processing, in order to isolate the desired coherence transfer pathways.

A. Preparation. An INEPT transfer from the initial longitudinal proton magnetization generates longitudinal two spin order:

$$I_z \rightarrow 2I_zS_z = I^\alpha S_z - I^\beta S_z$$

where the single transition operators $I^\alpha = E/2 + I_z$ and $I^\beta = E/2 - I_z$.

B. Spin-state selective excitation (S^3E). Transverse S magnetization is excited with phases dependent on the I spin state and the phase program, B_1–B_4, that is executed:

$$B_1\colon I^\alpha S_z \rightarrow -I^\beta S_x, \quad I^\beta S_z \rightarrow -I^\alpha S_y$$

$$B_2\colon I^\alpha S_z \rightarrow I^\beta S_x, \quad I^\beta S_z \rightarrow -I^\alpha S_y$$

$$B_3\colon I^\alpha S_z \rightarrow I^\beta S_y, \quad I^\beta S_z \rightarrow -I^\alpha S_x$$

$$B_4\colon I^\alpha S_z \rightarrow I^\beta S_y, \quad I^\beta S_z \rightarrow I^\alpha S_x$$

By forming linear combinations of subspectra acquired using these phase programs, the single quantum coherences $S^\pm = S_x \pm iS_y$ can be prepared and isolated in a spin-state selective manner

(where subspectra are denoted simply by their associated phase program, and multiplication by i indicates a 90º phase shift):

$$-B_1 + B_2 + i(B_3 + B_4): I^\alpha S_z \rightarrow \tfrac{1}{2} I^\beta S^+, \quad I^\beta S_z \rightarrow 0$$
$$-B_1 + B_2 - i(B_3 + B_4): I^\alpha S_z \rightarrow \tfrac{1}{2} I^\beta S^-, \quad I^\beta S_z \rightarrow 0$$
$$-B_3 + B_4 - i(B_1 + B_2): I^\alpha S_z \rightarrow 0, \quad I^\beta S_z \rightarrow \tfrac{1}{2} I^\alpha S^+$$
$$-B_3 + B_4 + i(B_1 + B_2): I^\alpha S_z \rightarrow 0, \quad I^\beta S_z \rightarrow \tfrac{1}{2} I^\alpha S^-$$

As relaxation pathways have been identical for all coherences prepared in this way, these states therefore serve as high quality starting points for the measurement of decay due to cross-correlated relaxation during step C. We note that all subspectra contribute constructively to each coherence, ensuring that sensitivity is maximized.

C. Relaxation and frequency encoding. Prepared coherences evolve for a constant time period *T*, during which chemical shifts are encoded for an evolution period t_1 with spin-state dependent frequencies ω_α and ω_β, and transverse relaxation occurs in a spin-state selective manner with rates $R_{2\alpha} = R_{2S} - \eta_{xy}$ and $R_{2\beta} = R_{2S} + \eta_{xy}$:

$$I^\alpha S^\pm \rightarrow \exp(\mp i\omega_\alpha t_1) \exp(-R_{2\alpha} T)\, I^\alpha S^\mp + \epsilon I^\beta S^\mp = \Omega_{\alpha\pm} R_\alpha I^\alpha S^\mp + \epsilon I^\beta S^\mp$$
$$I^\beta S^\pm \rightarrow \exp(\mp i\omega_\beta t_1) \exp(-R_{2\beta} T)\, I^\beta S^\mp + \epsilon I^\alpha S^\mp = \Omega_{\beta\pm} R_\beta I^\beta S^\mp + \epsilon I^\alpha S^\mp$$

where $\Omega_{\sigma\pm} = \exp(\mp i\omega_\sigma t_1)$ and $R_\sigma = \exp(-R_{2\sigma} T)$.

Proton spin-flips occurring during *T* give rise to terms in ϵ. However, provided that the rate of these spin flips $R_{1\mathrm{Hsel}} \ll |i2\pi J_{NH} + 2\eta_{xy}|$, these terms are non-secular and will be strongly suppressed (an effect that is optimized when the constant time period $T = n/J_{NH}$)[71].

D. Spin-state selective coherence transfer (S^{3}CT). I^α and I^β operators are transferred to $I^\pm$ operators (of which only I^+ terms will ultimately be observable). Following the first ^{1}H 90º pulse, a combination of multiple quantum and ^{15}N single quantum coherences are generated, which are fully converted to multiple quantum terms by the final ^{1}H 90º pulse. As these terms relax at different rates, care is required to obtain symmetrized combinations. Coherences evolve dependent upon the phase $\varphi_5 = \pm y$:

$$I^\alpha S^- \rightarrow -\tfrac{1}{2} i\left[\exp(-2R_{MQ}\delta) \mp \exp(-2R_{2S}\delta)\right] I^+ S^+ + \tfrac{1}{2} i\left[\exp(-2R_{MQ}\delta) \pm \exp(-2R_{2S}\delta)\right] I^- S^+$$
$$I^\alpha S^+ \rightarrow -\tfrac{1}{2} i\left[\exp(-2R_{MQ}\delta) \pm \exp(-2R_{2S}\delta)\right] I^+ S^- + \tfrac{1}{2} i\left[\exp(-2R_{MQ}\delta) \mp \exp(-2R_{2S}\delta)\right] I^- S^-$$
$$I^\beta S^- \rightarrow \tfrac{1}{2} i\left[\exp(-2R_{MQ}\delta) \pm \exp(-2R_{2S}\delta)\right] I^+ S^+ - \tfrac{1}{2} i\left[\exp(-2R_{MQ}\delta) \mp \exp(-2R_{2S}\delta)\right] I^- S^+$$
$$I^\beta S^+ \rightarrow \tfrac{1}{2} i\left[\exp(-2R_{MQ}\delta) \mp \exp(-2R_{2S}\delta)\right] I^+ S^- - \tfrac{1}{2} i\left[\exp(-2R_{MQ}\delta) \pm \exp(-2R_{2S}\delta)\right] I^- S^-$$

I^- terms will be transformed into unobservable I^+ coherences at the point of acquisition and can therefore be neglected in further discussions.

As the terms $A_{MQ} = \exp(-2R_{MQ}\delta)$ and $A_{2S} = \exp(-2R_{2S}\delta)$ are both expected to be close to one, for any given phase φ_5 efficient transfers (with amplitudes $\frac{1}{2}(A_{MQ} + A_{2S}) \approx 1$) can only be achieved for two of the four initial coherences. In order to observe all four coherences prepared at the end of part C (required to select both echo and anti-echo components of signals) it is therefore necessary to acquire separate subspectra, corresponding to the phase $\varphi_5 = \pm y$ (and

referred to as 1a/b and 2a/b). Interfering transfers from other coherences, shown here as terms in ζ, will be suppressed by a factor $\frac{A_{MQ}+A_{2S}}{A_{MQ}-A_{2S}} \gtrsim 20$ within these subspectra.

$$I^\alpha S^+ + \zeta I^\beta S^+ \xrightarrow{\phi_5=y} -\tfrac{1}{2}i\left[\left(A_{MQ}+A_{2S}\right)-\zeta\left(A_{MQ}-A_{2S}\right)\right]I^+S^- \approx -iI^+S^-$$

$$I^\beta S^- + \zeta I^\alpha S^- \xrightarrow{\phi_5=y} \tfrac{1}{2}i\left[\left(A_{MQ}+A_{2S}\right)-\zeta\left(A_{MQ}-A_{2S}\right)\right]I^+S^+ \approx iI^+S^+$$

$$I^\alpha S^- + \zeta I^\beta S^- \xrightarrow{\phi_5=-y} -\tfrac{1}{2}i\left[\left(A_{MQ}+A_{2S}\right)-\zeta\left(A_{MQ}-A_{2S}\right)\right]I^+S^+ \approx -iI^+S^+$$

$$I^\beta S^+ + \zeta I^\alpha S^+ \xrightarrow{\phi_5=-y} \tfrac{1}{2}i\left[\left(A_{MQ}+A_{2S}\right)-\zeta\left(A_{MQ}-A_{2S}\right)\right]I^+S^- \approx iI^+S^-$$

E. Spin-state selective coherence transfer (S^{3}CT). $S^\pm$ operators are transferred to S^α and S^β operators for detection as anti-TROSY and TROSY lines. Separate experiments, 1a/2a and 1b/2b, corresponding to a 180° phase shift of ψ_6, must be acquired in order to symmetrize the read-out of starting operators on both TROSY and anti-TROSY lines. A three-step phase cycle is also performed on ψ_6 and ψ_7 to select $\Delta p_S = +1$ or $\Delta p_S = -1$, and the necessary receiver phases (indicated above the arrows below) can be applied post-acquisition to isolate I^-S^α and I^-S^β subspectra.

$$I^+S^+ \xrightarrow{0°,120°,240°} -\tfrac{1}{2}\left(A_{MQ}+A_{2I}\right)I^-S^\alpha + \tfrac{1}{2}\left(A_{MQ}-A_{2I}\right)I^-S^\beta \approx -I^-S^\alpha$$

$$I^+S^- \xrightarrow{0°,240°,120°} -\tfrac{1}{2}\left(A_{MQ}+A_{2I}\right)I^-S^\beta + \tfrac{1}{2}\left(A_{MQ}-A_{2I}\right)I^-S^\alpha \approx -I^-S^\beta$$

$$I^+S^+ \xrightarrow{0°,120°,240°} \tfrac{1}{2}\left(A_{MQ}+A_{2I}\right)I^-S^\beta - \tfrac{1}{2}\left(A_{MQ}-A_{2I}\right)I^-S^\alpha \approx I^-S^\beta$$

$$I^+S^- \xrightarrow{0°,240°,120°} \tfrac{1}{2}\left(A_{MQ}+A_{2I}\right)I^-S^\alpha - \tfrac{1}{2}\left(A_{MQ}-A_{2I}\right)I^-S^\beta \approx I^-S^\alpha$$

Following the first ^{15}N 90° pulse, a combination of multiple quantum and ^{1}H single quantum coherences are generated, which are fully converted to ^{1}H single quantum terms by the final ^{15}N 90° pulse. Accounting for the differential relaxation of these terms, we have the transfer amplitudes $A_{MQ} = exp\ exp\left(-2R_{MQ}\delta\right)$ and $A_{2I} = exp\left(-2R_{2I}\delta\right)$. Thus, we have obtained relaxation-symmetrized detection of $I^+S^\pm$ coherences as both I^-S^α and I^-S^β transitions, in which transfer to the other component of the doublet is suppressed by a factor $\frac{A_{MQ}+A_{2I}}{A_{MQ}-A_{2I}} \gtrsim 20$.

F. Recovery. During the back-transfer steps D and E, I_z magnetization recovered by longitudinal relaxation during the relaxation period C is transformed into $\pm S_z$ magnetization. Immediately following acquisition of the FID, ^{15}N pulses are applied where necessary (depending on the phases ϕ_5 and ψ_6) to invert $-S_z$ magnetization to $+S_z$, to ensure that longitudinal relaxation and cross-correlated relaxation are as similar as possible between adjacent FIDs.

Implementation. A Bruker format pulse sequence for the experiment is available online (https://github.com/chriswaudby/pp/blob/master/best_trosy_ccr.cw) and in the Source Data , together with nmrPipe and Python scripts (https://github.com/chriswaudby/pp/blob/master/scripts/proc-15n-ccr.com and

https://github.com/chriswaudby/pp/blob/master/scripts/proc-15n-ccr.py) to perform the necessary processing.

Supplementary References

63. Lee, D., Hilty, C., Wider, G. & Wüthrich, K. Effective rotational correlation times of proteins from NMR relaxation interference. *Journal of Magnetic Resonance* **178,** 72–76 (2006).
64. Williamson, M. P. Using chemical shift perturbation to characterise ligand binding. *Prog Nucl Magn Reson Spectrosc* **73,** 1–16 (2013).
65. Dehouck, Y., Kwasigroch, J. M., Gilis, D. & Rooman, M. PoPMuSiC 2.1: a web server for the estimation of protein stability changes upon mutation and sequence optimality. *BMC Bioinformatics* **12,** 151–151 (2011).
66. Roehrl, M. H. A., Heffron, G. J. & Wagner, G. Correspondence between spin-dynamic phases and pulse program phases of NMR spectrometers. *Journal of Magnetic Resonance* **174,** 325–330 (2005).
67. Pelupessy, P., Espallargas, G. M. & Bodenhausen, G. Symmetrical reconversion: measuring cross-correlation rates with enhanced accuracy. *Journal of Magnetic Resonance* **161,** 258–264 (2003).
68. Hall, J. B., Dayie, K. T. & Fushman, D. Direct measurement of the 15N CSA/dipolar relaxation interference from coupled HSQC spectra. *J Biomol NMR* **26,** 181–186 (2003).
69. Vasos, P. R., Hall, J. B. & Fushman, D. Spin-state selection for increased confidence in cross-correlation rates measurements. *J Biomol NMR* **31,** 149–154 (2005).
70. Rennella, E. & Brutscher, B. Fast Real-Time NMR Methods for Characterizing Short-Lived Molecular States. *Chemphyschem* **14,** 3059–3070 (2013).
71. Ghose, R. & Prestegard, J. H. Improved Estimation of CSA–Dipolar Coupling Cross-Correlation Rates from Laboratory-Frame Relaxation Experiments. *Journal of Magnetic Resonance* **134,** 308–314 (1998).
