# Supplementary figures and images for: Interactions between nascent proteins and the ribosome surface inhibit co-translational folding
Source: Nat Chem. 2021 Oct 14;13(12):1214–20. doi: 10.1038/s41557-021-00796-x (PMC8627912; doi:10.1038/s41557-021-00796-x)

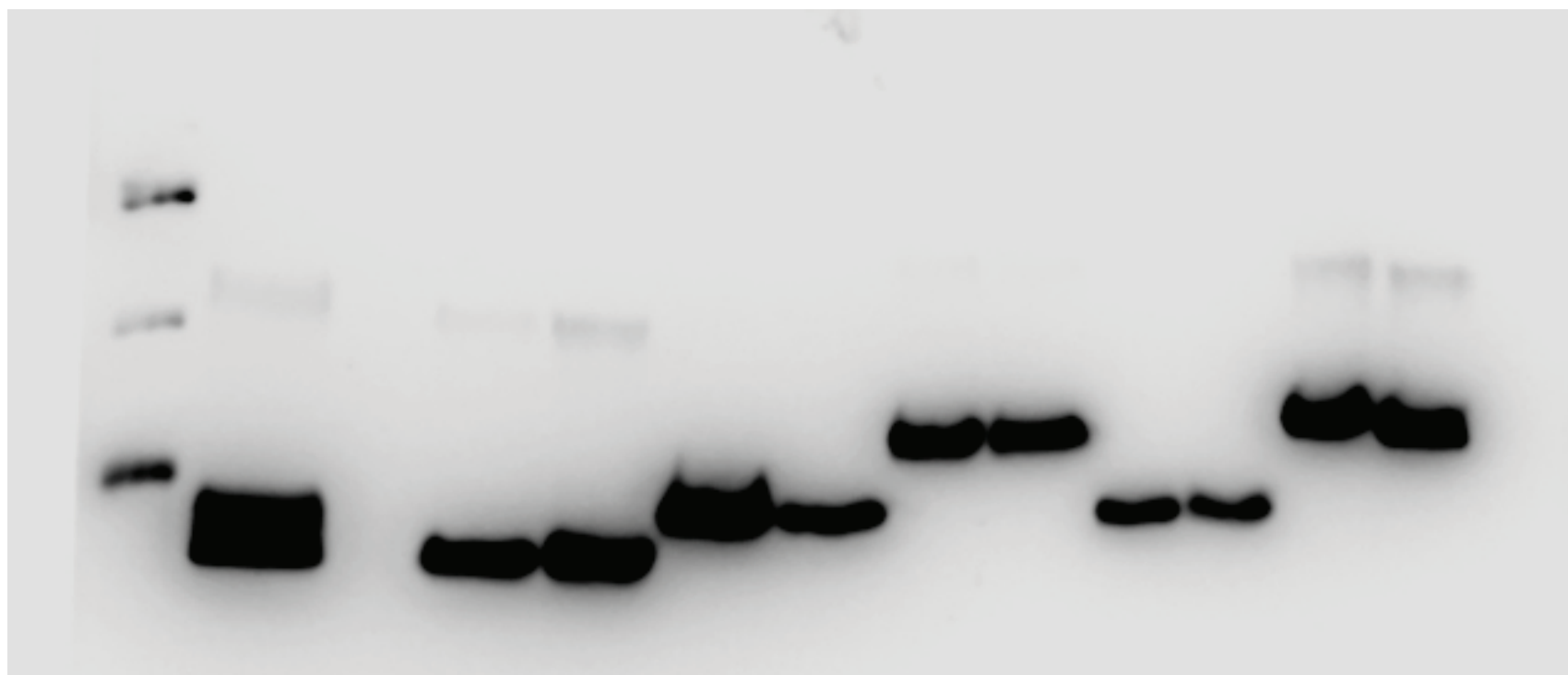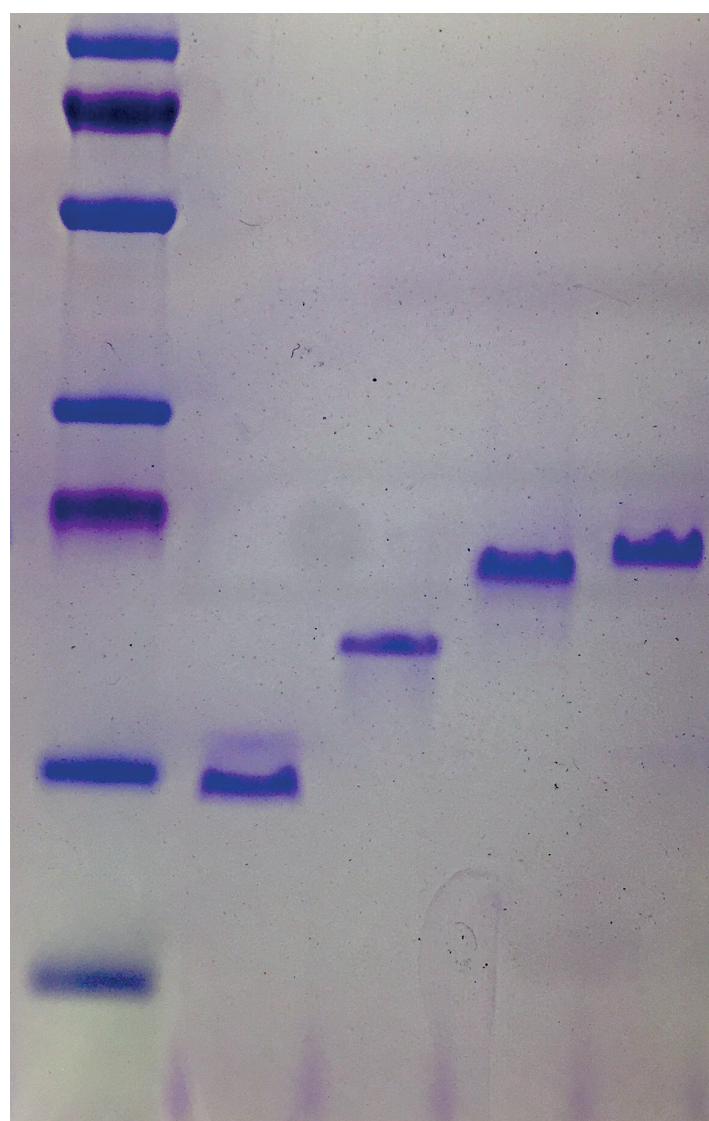

Supplement: Source Data Extended Data Fig. 2 — Unprocessed gel data [file 41557_2021_796_MOESM9_ESM.pdf]

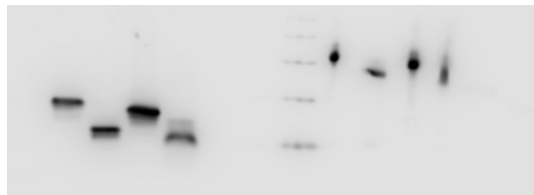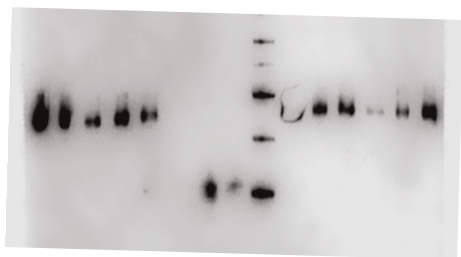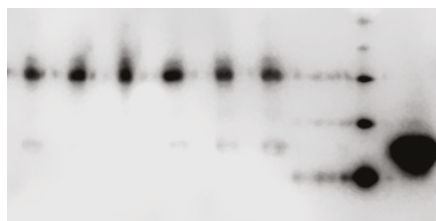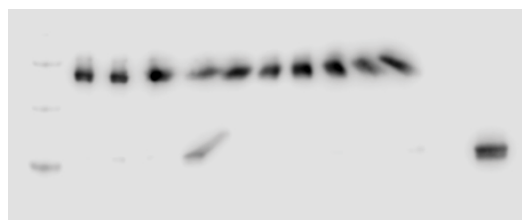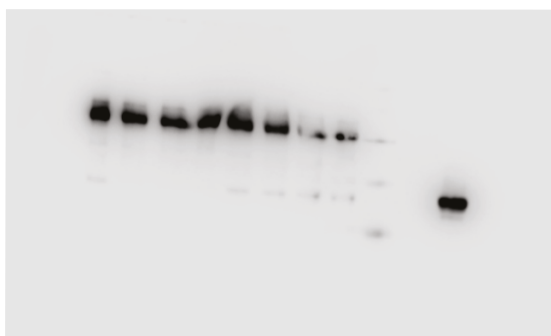

Supplement: Source Data Extended Data Fig. 3 — Unprocessed gel data [file 41557_2021_796_MOESM11_ESM.pdf]

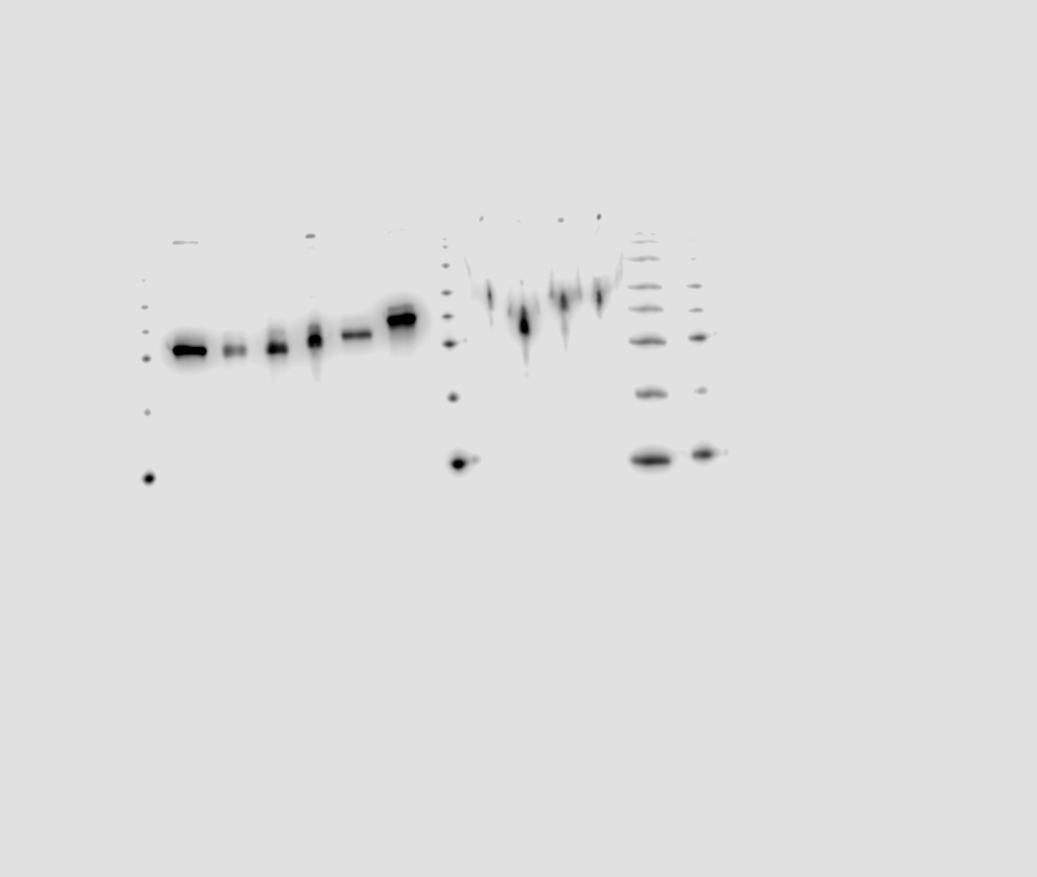

Supplement: Source Data Extended Data Fig. 5 — Unprocessed gel data [file 41557_2021_796_MOESM15_ESM.jpg]

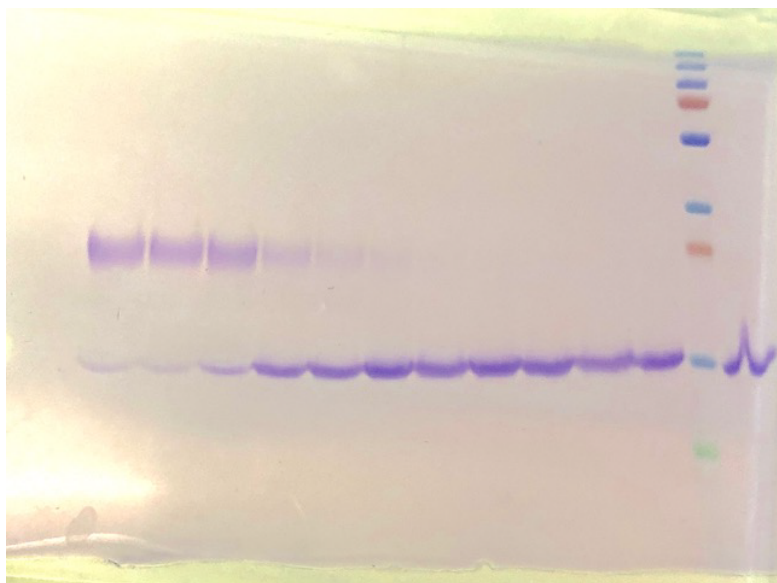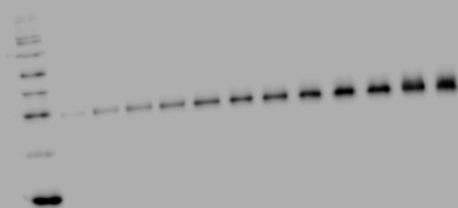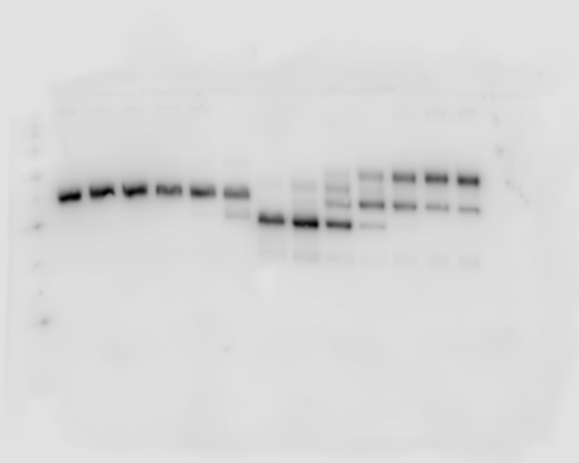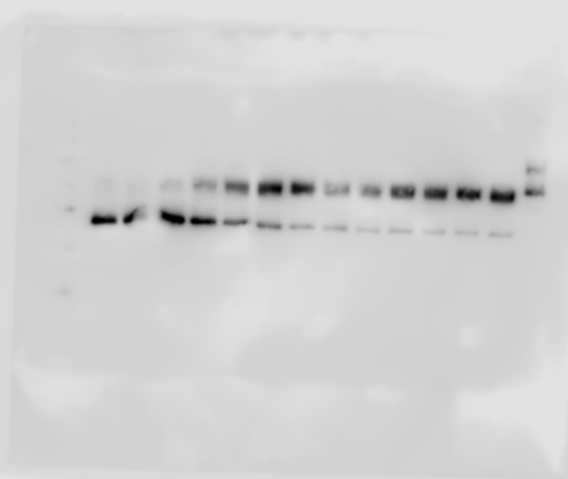

Supplement: Source Data Extended Data Fig. 10 — Unprocessed gel data [file 41557_2021_796_MOESM21_ESM.pdf]
