# Supplementary material for: Interactions between nascent proteins and the ribosome surface inhibit co-translational folding
Source: Nat Chem. 2021 Oct 14;13(12):1214–20. doi: 10.1038/s41557-021-00796-x (PMC8627912; doi:10.1038/s41557-021-00796-x)
Supplement: Source Data Extended Data Fig. 4 — Source code [file 41557_2021_796_MOESM13_ESM.pdf]

BEST TROSY CCR: pulse sequence

```
;BEST_TROSY_CCR
;
;improved phase cycling
;eliminating the D1 3-step phase cycle
;
;Chris Waudby, Mar 2016
;
;based on BT_TRACT
;
;(E. Lescop, P. Schanda & B. Brutscher,
;   J. Magn. Reson.  187 163-169 (2007))
;
;$CLASS=
;$DIM=2D
;$TYPE=
;$SUBTYPE=
;$COMMENT=


prosol relations=<triple>

#include <Avance.incl>
#include <Grad.incl>
#include <Delay.incl>


/*******************************************************/
/*  Predefined shapes for 1H pulses       *************/
/*  cnst1: center of excitation band     **************/
/*  cnst2: excitation band width         **************/
/*******************************************************/

/*  PC9 (p41, sp25)    */
"p41=7.2/(cnst2*bf1/1000000)" /*  PC9  pulse length  */
"spoff25=bf1*(cnst1/1000000)-o1"  /*  PC9  offset */

/*  REBURP (p42, sp26)   */

"p42=1.08*4.875/(cnst2*bf1/1000000)" /* REBURP pulse length  */
"spoff26=bf1*(cnst1/1000000)-o1" /* REBURP offset */

/*  EBURP & EBURP_TR  (p43, sp28, sp29)   */

"p43=4.6/(cnst2*bf1/1000000)" /*  EBURP pulse length   */
"spoff28=bf1*(cnst1/1000000)-o1" /*  EBURP offset */
"spoff29=bf1*(cnst1/1000000)-o1" /*  EBURP_REV offset */


/*******************************************************/
/*  Gradient pulse durations              *************/
/*******************************************************/

;"p16=1000u"
;"p17=300u"


/*********************************************************************/
/*   DELAYS                                                          */
/*********************************************************************/

"d11=30m"

"d25=2.77m"
"d26=2.77m"
"d27=2.77m"  /* set slightly shorter than d26 for relaxation compensation  */
"d28=0.5*d26" ; 1/8J


"DELTA1=d25-p41*0.5-p42*0.5-p17-4u"
"DELTA2=d27-p17-d16-p42*0.5-p43*0.5"
"DELTA3=d26-p17-d16-p42*0.5"
```

```
"DELTA4=d28-p42*0.5-p17-4u"

"d3=p21*2/3.1416"
"p22=2*p21"
"d22=p22"

"d31=d1-de-aq-d11-76u-2*p21-p16-d16"

/*********************************************************************/
/*   time increments in 15N dimension                              */
/*********************************************************************/
"in0=inf2*0.5"
"in10=inf2*0.5"

"d0=0.5*d20-p21-p21*2/3.1416-p16-d16"
"d10=0.5*d20-p21-4u-p16-d16"

;"l0=1"  ; flag for 15N inversion at start of sequence
;"l1=1"  ; flag for dummy scans
;"l31=1" ; flag for 15N inversion at end of sequence

"acqt0=d3-4u-p21"

aqseq 312




/*****************************************************************************/
/*   START DUMMY SCANS                                                       */
/*****************************************************************************/

11 ze
12 30u
13 4u
14 d11
  38u
15 4u pl0:f1 pl3:f3

d31 ; relaxation delay

; invert equilibrium Sz
(p21 ph10):f3
2u
(p21 ph1):f3

4u UNBLKGRAD
p16:gp1
d16

/***************************************/
/*   H-N transfer (DUMMY SCANS)       */
/***************************************/
(p41:sp25 ph29):f1    /*  PC9  */
4u
p17:gp2
DELTA1
(center (p42:sp26 ph10) (p22 ph10):f3 )
4u
p17:gp2
DELTA1
(p41:sp25 ph11):f1   /*  PC9  */

p16:gp3  ; zz filter
d16

/***************************************/
/*   S3E element (propagator B) (DS)  */
/***************************************/
(p21 ph2):f3
4u
p17:gp4
DELTA4   ; 1/8J
(center (p42:sp26 ph23) (p22 ph20):f3 )  ; (pi) I(-45deg) + (pi) S(-45deg)
                                          ; ph23 = 315deg, ph20=45deg (bruker phases)
4u
p17:gp4
```

```
DELTA4   ; 1/8J
(p21 ph11):f3  ; (pi/2)S-y -> (pi/2)Sy (bruker phases)
2u
(p21 ph3):f3


/*******************************************/
/*   15N relaxation and shift evolution  */
/*   (propagator C)  (DUMMY SCANS)       */
/*******************************************/
d0
p16:gp5
d16
(p22 ph10):f3
4u
p16:gp5
d16
d10

/****************************************/
/*   S3CT (propagator D part 1) (DS)   */
/****************************************/
(p43:sp29 ph4):f1   /* EBURP_REV */
p17:gp6
d16
DELTA2
(center (p42:sp26 ph5):f1 (p22 ph10):f3 )
DELTA2
p17:gp6
d16

(ralign (p43:sp28 ph6 d3):f1 (p21 ph7):f3 )  /* EBURP  */

/****************************************/
/*   S3CT (propagator D part 2) (DS)   */
/****************************************/

p17:gp7
d16
DELTA3
(center (p42:sp26 ph10) (p22 ph8):f3 )
DELTA3
p17:gp7
d16
(p21 ph9):f3
4u BLKGRAD

/****************************************/
/*   "Signal detection" (DUMMY SCANS) */
/****************************************/
  aq
  de

; invert Sz (for better recovery of unused magnetisation)
  4u
  (p21 ph12 p21 ph9):f3

/****************************************/
/*   Looping (DUMMY SCANS)             */
/****************************************/
4u ippall ; increment all phase programs

; axial peak suppression
lo to 12 times 2

; store data and move to next buffer
30u ;st
lo to 13 times nbl

4u rppall
lo to 14 times ds




/****************************************************************************/
```

```
/*   START DATA ACQUISITION                                                */
/*****************************************************************************/
;30u st0

1 zd
2 30u
3 4u
4 d11
  38u
5 4u pl0:f1 pl3:f3

  d31 ; relaxation delay

  ; invert equilibrium Sz
  (p21 ph10):f3
  2u
  (p21 ph1):f3

  4u UNBLKGRAD
  p16:gp1
  d16

/***************************************/
/*   H-N transfer                      */
/***************************************/
  (p41:sp25 ph29):f1    /*  PC9  */
  4u
  p17:gp2
  DELTA1
  (center (p42:sp26 ph10) (p22 ph10):f3 )
  4u
  p17:gp2
  DELTA1
  (p41:sp25 ph11):f1   /*  PC9  */

  p16:gp3  ; zz filter
  d16

/***************************************/
/*   S3E element (propagator B)        */
/***************************************/
  (p21 ph2):f3
  4u
  p17:gp4
  DELTA4   ; 1/8J
  (center (p42:sp26 ph23) (p22 ph20):f3 )  ; (pi) I(-45deg) + (pi) S(-45deg)
                                           ; ph23 = 315deg, ph20=45deg (bruker phases)
  4u
  p17:gp4
  DELTA4   ; 1/8J
  (p21 ph11):f3  ; (pi/2)S-y -> (pi/2)Sy (bruker phases)
  2u
  (p21 ph3):f3

/******************************************/
/*   15N relaxation and shift evolution  */
/*   (propagator C)                       */
/******************************************/
  d0
  p16:gp5
  d16
  (p22 ph10):f3
  4u
  p16:gp5
  d16
  d10

/***************************************/
/*   S3CT (propagator D part 1)        */
/***************************************/
  (p43:sp29 ph4):f1   /* EBURP_REV */
  p17:gp6
  d16
  DELTA2
  (center (p42:sp26 ph5):f1 (p22 ph10):f3 )
  DELTA2
  p17:gp6
```

```
  d16

(ralign (p43:sp28 ph6 d3):f1 (p21 ph7):f3 )  /* EBURP  */

/***************************************/
/*   S3CT (propagator D part 2)       */
/***************************************/

  p17:gp7
  d16
  DELTA3
  (center (p42:sp26 ph10) (p22 ph8):f3 )
  DELTA3
  p17:gp7
  d16
  (p21 ph9):f3
  4u BLKGRAD

/***************************************/
/*   Signal detection                 */
/***************************************/
  goscnp ph31

; invert Sz (for better recovery of unused magnetisation)
  4u
  (p21 ph12 p21 ph30):f3

/***************************************/
/*   Looping                          */
/***************************************/
4u ippall ; increment all phase programs

; axial peak suppression
lo to 2 times 2

; store data and move to next buffer
30u st
lo to 3 times nbl

4u rppall
lo to 4 times ns

; save buffer contents to disk
d11 wr #0 if #0
30u zd

; t1 evolution
4u dd0
4u id10
lo to 5 times l2

exit



ph1=0 0 2 2
ph2=(8) {7 3}*2 {1 5}*2          ; (45 225)*2 (315 135)*2 (bruker 15N phases)
ph3={1}*8 {3}*8                  ; (-y)8 (y)8 (bruker 15N phases)
ph4=(12) 0 ;{0}*16 {4}*16 {8}*16    ; 0deg 120deg 240deg
ph5=(12) 0 ;{0}*16 {4}*16 {8}*16    ; 0deg 120deg 240deg
ph6=(12) {3}*16 {9}*16 ; {3}*16 {7}*16 {11}*16 {9}*16 {1}*16 {5}*16  ; 90deg 270deg
ph7=(12) {9}*32 {5}*32 {1}*32 {3}*32 {11}*32 {7}*32  ; 90deg 210deg 330deg 270deg 30deg 150deg
(bruker 15N phases)
ph8=(12) {0}*32 {8}*32 {4}*32    ; 0deg 120deg 240deg (bruker 15N phases)
ph9=(12) {0}*32 {8}*32 {4}*32    ; 0deg 120deg 240deg (bruker 15N phases)

ph29={0}*192 {2}*192
ph30={{2}*16 {0}*16}*3 {{0}*16 {2}*16}*3   ; for recovery of Sz magnetization, check sign
ph31=0 2   ; axial peak suppression

ph10=0
ph11=1
ph12=2
ph13=3
ph20=(8) 1
ph21=(8) 3
ph22=(8) 5
```

```
ph23=(8) 7

;pl0 : 0W
;pl1 : f1 channel - power level for pulse (default)
;pl3 : f3 channel - power level for pulse (default)
;sp25: Pc9_4_90.1000
;sp26: Reburp.1000
;sp28: Eburp2.1000
;sp29: Eburp2tr.1000
;sp20: f3 channel - BIP
;sp21: f3 channel - Reburp
;p16:  gradient pulse                        [1 msec]
;p17:  gradient pulse                        [300 usec]
;p21: f3 channel -  90 degree high power pulse
;p22: f3 channel - 180 degree high power pulse
;p41: PC9
;p42: REBURP
;p43: EBURP2
;d0 : incremented delay (F1)                          [3 usec]
;d1 : relaxation delay; 1-5 * T1
;d11: delay for disk I/O                              [30 msec]
;d16: delay for homospoil/gradient recovery
;d20: constant time 15N relaxation/evolution period
;d25: 1/(4J(NH)
;d26: 1/(4J(NH)
;d27: 1/(4J(NH)
;cnst1: H(N) excitation frequency (in ppm)
;cnst2: H(N) excitation band width (in ppm)
;cnst26: Call chemical shift (offset, in ppm)          [101 ppm]
;cnst41: Power change for PC9 pulse (dB)
;cnst42: Power change for REBURP pulse (dB)
;cnst43: Power change for EBURP2 pulse (dB)
;inf1: 1/SW(N) = 2 * DW(N)
;in0: 1/(2 * SW(N)) = DW(N)
;nd0: 2
;ns: 1 * n
;ds: >= 1
;NBL: 192
;td1: 96, number of experiments in F1 per REAL point
;td2: number of REAL points in F2 (15N)
;l2: number of COMPLEX points
;FnMODE: echo-antiecho in F1


;for z-only gradients:
;gpz1: 31.4% (Nz purge)
;gpz2: 23% (INEPT)
;gpz3: 21% (zz filter)
;gpz4: 31% (S3E)
;gpz5: 11% (15N CT refocusing)
;gpz6: 16.7% (S3CT 1)
;gpz7: 45% (S3CT 2)

;use gradient files:
;gpnam1: SMSQ10.100
;gpnam2: SMSQ10.32
;gpnam3: SMSQ10.100
;gpnam4: SMSQ10.32
;gpnam5: SMSQ10.100
;gpnam6: SMSQ10.32
;gpnam7: SMSQ10.32
```

BEST TROSY CCR: processing scripts

Shell script (which calls python script, below)

```
#!/bin/csh

# NB process as 2D (ymod complex)
./fid.com

# split into components
python proc-15n-ccr.py

# process each component
```

```
set expts = (naa nab nba nbb haa hab hba hbb)

foreach i (`seq 8`)

echo {$expts[$i]}.fid

nmrPipe -in {$expts[$i]}.fid \
| nmrPipe -fn SOL                                        \
| nmrPipe  -fn SP -off 0.5 -end 1.00 -pow 2 -c 0.5       \
| nmrPipe  -fn ZF -zf 2                                  \
| nmrPipe  -fn FT -auto                                  \
| nmrPipe  -fn PS -p0 -156.00 -p1 123.00 -di -verb       \
| nmrPipe  -fn EXT -x1 7.5ppm -xn 9ppm -sw               \
| nmrPipe  -fn TP                                        \
| nmrPipe  -fn LP -fb -ps0-0                             \
| nmrPipe  -fn SP -off 0.5 -end 1.00 -pow 2 -c 0.5       \
| nmrPipe  -fn ZF -zf 1                                  \
| nmrPipe  -fn FT -auto                                  \
| nmrPipe  -fn PS -p0 0.00 -p1 0.00 -di -verb            \
| nmrPipe  -fn TP                                        \
   -ov -out {$expts[$i]}.ft2

rm {$expts[$i]}.fid

end
```

Python script (proc-15n-ccr.py):

```
/usr/bin/env python

import sys
import nmrglue as ng
import numpy as np
from copy import deepcopy

propagators = {
    'IaS+ -> a' : {'B':4, 'D1':'sum','D2':'+a'},
    'IaS+ -> b' : {'B':4, 'D1':'sum','D2':'+b'},
    'IaS- -> a' : {'B':3, 'D1':'diff','D2':'-a'},
    'IaS- -> b' : {'B':3, 'D1':'diff','D2':'-b'},
    'IbS+ -> a' : {'B':2, 'D1':'diff','D2':'+a'},
    'IbS+ -> b' : {'B':2, 'D1':'diff','D2':'+b'},
    'IbS- -> a' : {'B':1, 'D1':'sum','D2':'-a'},
    'IbS- -> b' : {'B':1, 'D1':'sum','D2':'-b'}
}

#expt = str(sys.argv[1])

def main():
    process_component('IbS+ -> b','IbS- -> b',output_filename='hbb.fid')
    process_component('IaS+ -> b','IaS- -> b',output_filename='hab.fid')
    process_component('IbS+ -> a','IbS- -> a',output_filename='hba.fid')
    process_component('IaS+ -> a','IaS- -> a',output_filename='haa.fid')

    process_component('IbS+ -> b','IbS- -> b',output_filename='nbb.fid',start='N')
    process_component('IaS+ -> b','IaS- -> b',output_filename='nab.fid',start='N')
    process_component('IbS+ -> a','IbS- -> a',output_filename='nba.fid',start='N')
    process_component('IaS+ -> a','IaS- -> a',output_filename='naa.fid',start='N')



def process_component(prop1, prop2, output_filename, start='H'):
    # import the raw FID data
    #dic,data=ng.pipe.read(expt + '/test.fid')
    dic,data=ng.pipe.read('test.fid')
    udic = ng.pipe.guess_udic(dic, data)    # set the spectral parameters

    dic_echo, data_echo = isolate_propagator(udic, data, start=start,
pathway=propagators[prop1])
    dic_antiecho, data_antiecho = isolate_propagator(udic, data, start=start,
pathway=propagators[prop2])

    # resolve echo/anti-echo components
    output_dic = deepcopy(dic_echo)
    output_dic[0]['size'] *= 2
    output_data = np.zeros((output_dic[0]['size'],output_dic[1]['size']),dtype='complex64')
```

```
    output_data[::2] = data_echo + data_antiecho
    output_data[1::2] = 1j*(data_echo - data_antiecho)

    if start=='N' and 'Ia' in prop1:
        # reverse I(alpha) terms in N-start experiments
        output_data *= -1

    # write processed data back to nmrPipe by directly editing original dictionary
    dic['FDF1APOD'] //= 96
    dic['FDF1TDSIZE'] //= 96
    dic['FDSPECNUM'] //= 96

    #ng.pipe.write(expt + '/' + output_filename, dic, output_data, overwrite=True)
    ng.pipe.write(output_filename, dic, output_data, overwrite=True)

    # # write the processed data back to nmrPipe format (via universal dictionary)
    # C = ng.convert.converter()
    # C.from_universal(output_dic, output_data)
    # ng.pipe.write(expt + '/' + output_filename, *C.to_pipe(), overwrite=True)


def isolate_propagator(input_dic, input_data, start, pathway):
    # start = 'H' or 'N'
    # pathway = dictionary with B, D1 and D2
    # B = 1 to 4
    # D1 = 'sum' or 'diff'
    # D2 = '+a', '+b', '-a' or '-b'
    B = pathway['B']
    D1 = pathway['D1']
    D2 = pathway['D2']

    # make a copy of the input dictionary for the processed data
    dic = deepcopy(input_dic)

    # split into H-start and N-start
    dataH = input_data[::2] + input_data[1::2]
    dataN = input_data[::2] - input_data[1::2]

    # select H-start or N-start to work with...
    if start == 'H':
        data = dataH
    else:
        data = dataN
    dic[0]['size'] //= 2     # update spectrum size

    # make linear combinations of B propagators
    # dataB will become a list with the four combinations
    if B == 1:
        data = -data[0::4,:]+data[2::4,:]+1j*(data[1::4,:]+data[3::4,:])
    elif B == 2:
        data = -data[0::4,:]+data[2::4,:]-1j*(data[1::4,:]+data[3::4,:])
    elif B == 3:
        data = -data[1::4,:]+data[3::4,:]-1j*(data[0::4,:]+data[2::4,:])
    else:
        data = -data[1::4,:]+data[3::4,:]+1j*(data[0::4,:]+data[2::4,:])
    dic[0]['size'] //= 4     # update spectrum size

    # phase cycles for first S3CT propagators
    #psi1 = [0., 240., 120., 0., 240., 120.]
    #psi2 = [0., 240., 120., 180., 60., 300.]
    data1 = np.copy(data)
    data2 = np.copy(data)
    #for i in range(6):
    #    data1[i::6] *= np.exp( 1j * np.pi * psi1[i] / 180. )
    #    data2[i::6] *= np.exp( 1j * np.pi * psi2[i] / 180. )
    data2[1::2] *= -1

    if D1 == 'diff':
        data = data1 + data2
    else:
        data = data1 - data2
    data = data[0::2] + data[1::2] # + data[2::6] + data[3::6] + data[4::6] + data[5::6]
    dic[0]['size'] //= 2     # update spectrum size

    # phase cycles for second S3CT propagators
    psi1 = [0., 120., 240., 0., 120., 240.]
    psi2 = [0., 240., 120., 0., 240., 120.]
```

```
    psi3 = [0., 120., 240., 180., 300., 60.]
    psi4 = [0., 240., 120., 180., 60., 300.]
    data1 = np.copy(data)
    data2 = np.copy(data)
    data3 = np.copy(data)
    data4 = np.copy(data)
    for i in range(6):
        data1[i::6] *= np.exp( 1j * np.pi * psi1[i] / 180. )
        data2[i::6] *= np.exp( 1j * np.pi * psi2[i] / 180. )
        data3[i::6] *= np.exp( 1j * np.pi * psi3[i] / 180. )
        data4[i::6] *= np.exp( 1j * np.pi * psi4[i] / 180. )
    if D2 == '+b':
        data = data1 - data3
    elif D2 == '+a':
        data = -1 * (data1 + data3)
    elif D2 == '-b':
        data = -1 * (data2 + data4)
    elif D2 == '-a':
        data = data2 - data4
    data = data[0::6] + data[1::6] + data[2::6] + data[3::6] + data[4::6] + data[5::6]
    dic[0]['size'] //= 6     # update spectrum size

    # finally combine H start phase cycling
    if start == 'H':
        data = data[0::2] - data[1::2]
    else:
        data = data[0::2] + data[1::2]

    dic[0]['size'] //= 2     # update spectrum size


    return dic, data


main()
```
